# Differences in F pocket impact on HLA I genetic associations with autoimmune diabetes

**DOI:** 10.3389/fimmu.2024.1342335

**Published:** 2024-03-25

**Authors:** Xu Ren, A. W. Peshala Amarajeewa, M. D. Tharushika Jayasinghe, Malgorzata A. Garstka

**Affiliations:** ^1^ Department of Urology, Second Affiliated Hospital of Xi’an Jiaotong University, Xi’an, Shaanxi, China; ^2^ Core Research Laboratory, Second Affiliated Hospital of Xi’an Jiaotong University, Xi’an, Shaanxi, China; ^3^ Department of Endocrinology, Second Affiliated Hospital of Xi’an Jiaotong University, Xi’an, Shaanxi, China; ^4^ Department of Tumor and Immunology, Precision Medical Institute, Western China Science and Technology Innovation Port, Second Affiliated Hospital of Xi’an Jiaotong University, Xi’an, Shaanxi, China

**Keywords:** HLA class I, antigen presentation, peptide binding, polymorphism, inflammation, autoimmune diabetes

## Abstract

**Introduction:**

Human leukocyte antigen (HLA) I molecules present antigenic peptides to activate CD8^+^ T cells. Type 1 Diabetes (T1D) is an auto-immune disease caused by aberrant activation of the CD8^+^ T cells that destroy insulin-producing pancreatic β cells. Some *HLA I* alleles were shown to increase the risk of T1D (T1D-predisposing alleles), while some reduce this risk (T1D-protective alleles).

**Methods:**

Here, we compared the T1D-predisposing and T1D-protective allotypes concerning peptide binding, maturation, localization and surface expression and correlated it with their sequences and energetic profiles using experimental and computational methods.

**Results:**

T1D-predisposing allotypes had more peptide-bound forms and higher plasma membrane levels than T1D-protective allotypes. This was related to the fact that position 116 within the F pocket was more conserved and made more optimal contacts with the neighboring residues in T1D-predisposing allotypes than in protective allotypes.

**Conclusion:**

Our work uncovers that specific polymorphisms in HLA I molecules potentially influence their susceptibility to T1D.

## Introduction

Human leukocyte antigen (HLA) class I molecules are important components of the adaptive immune system. HLA I molecules are assembled from heavy chain, beta_2_ microglobulin (β_2_m) and antigenic peptides translocated from the cytosol to the endoplasmic reticulum (ER) by the transporter associated with antigen processing (TAP). Peptides bind to HLA I molecules through interactions with the peptide-binding groove (1). The assembled peptide-HLA I complexes are then transported through the Golgi apparatus to the cell surface to present antigens to CD8^+^ T cells (1). Upon recognizing a peptide-HLA I complex, CD8^+^ T cells become activated, secrete cytokines, including interferon-gamma (IFN-γ), and kill the antigen-presenting cell (2). At the cell surface, peptide and β_2_m eventually dissociate from the HLA I heavy chain, and peptide-free forms are endocytosed and degraded in the endolysosomal compartments (3).

Type 1 diabetes (T1D) is a disease caused by aberrant activation of the immune system, becoming a pervasive worldwide problem (4). In T1D, CD8^+^ T cells recognize auto-antigens presented by HLA I on pancreatic β cells, resulting in β cell destruction (2). Besides, HLA I molecules are hyper-expressed on pancreatic β cells (4). HLA class II molecules are long known to have a genetic association with T1D. Still, these genes cannot completely rationalize the link between HLA and T1D. *HLA-A, B*, and *C* genotypes were identified as the risk factors for T1D after accounting for linkage disequilibrium for T1D-associated HLA class II haplotypes (5–7). Certain HLA I alleles have been demonstrated to increase or decrease T1D risk (5–9). T1D predisposing alleles include but are not limited to, A*02:01 (5, 8), HLA-A*24:02 (5, 6, 8, 9), B*39:01 (21, 22), HLA-B*39:06 (5, 7, 8), B*44:05 (5), while protective alleles are comprised of HLA-A*11:01 (5, 6, 8, 9), HLA-B*38:01 (6, 8), HLA-B*44:02 (5, 8), HLA-B*44:03 (5, 7, 8), and HLA-B*57:01 (5, 7, 8).

It is not well understood what makes a particular *HLA I* allele T1D-predisposing or protective. HLA I molecules are highly polymorphic, and polymorphic residues are primarily located in the peptide-binding groove that may affect peptide selection and presentation (10). Polymorphisms within the peptide binding groove were shown to govern the association of HLA-B*27 alleles with ankylosing spondylitis (11), HLA-A*29:02 with Birdshot uveitis, HLA-B*51 with Behçet disease and HLA-C*06:02 with psoriasis (12). Presentation of self, immunogenic peptides by HLA I molecules that trigger autoimmune responses has been suggested as a primary mechanism driving these diseases (12). We hypothesized that the genetic polymorphism in T1D-associated HLA I molecules, which defines their structural features and function, may promote self-antigen binding and presentation, resulting in aberrant recognition by CD8^+^ T cells and autoimmunity. Here, we systematically compared T1D-predisposing and protective allotypes through a combination of cellular, biochemical, and computational approaches. Specifically, we analyzed HLA I surface expression, maturation status, and intracellular localization under normal, inflammatory, and peptide-limiting conditions. We analyzed HLA I peptide binding and the quality of peptidomes. Finally, by comparing sequences and energetic profiles in T1D-associated HLA I allotypes, we observed better energetic adaptation of residues in the F pocket to the surrounding residues in T1D-predisposing allotypes. We identified positions 114 and 116 as critical determinants of this adaptation, which might be essential for the stability of HLA I molecules.

## Materials and methods

### DNA constructs

A*02:01 (IMGT/HLA No.HLA00005), A*24:02 (IMGT/HLA No.HLA00050), A*11:01 (IMGT/HLA No.HLA00043) and B*57:01 (IMGT/HLA No.HLA00381) were a kind gift from Jacques Neefjes (Leiden University Medical Center, the Netherlands). B*44:02 (IMGT/HLA No.HLA00318) and B*44:05 (IMGT/HLA No.HLA00322) were a kind gift from Sebastian Springer (Constructor University, Bremen, Germany). B*44:03 (IMGT/HLA No.HLA00319) was generated by site-directed mutagenesis using B*44:02 as a template and confirmed by sequencing (Sangon, China. B*39:06 (IMGT/HLA No.HLA00279) and B*38:01 (IMGT/HLA No.HLA00267) genes were synthesized, inserted into a pMA-T vector and verified by sequencing (Invitrogen, USA). B*39:01 (IMGT/HLA No.HLA00271) was generated by site-directed mutagenesis using B*39:06 as a template and confirmed by sequencing (Sangon, China).

A*02:01, A*24:02, B*39:01, B*39:06, B*44:05, A*11:01, B*38:01, B*44:02, B*44:03 and B*57:01 heavy chains were cloned into pMX-GFP-N1 vector backbone (a kind gift from Jacques Neefjes, Leiden University Medical Center, the Netherlands, and Robbert Spaapen, at the time at Sanquin Blood Bank, Amsterdam, the Netherlands) via the EcoRI and BamHI restriction sites. Where mentioned, N-terminal HA-tagged versions of HLA I allotypes in the same pMX-GFP-N1 vector were used. HA epitope tag was introduced after the signal sequence by QuikChange site-directed mutagenesis (Agilent, CA, USA). A*02:01 Y116D and B*57:01 S116Y mutants were purchased from Genewiz (Suzhou, China). All the constructs were confirmed by DNA sequencing (Sangon, Shanghai, China).

### Cell lines and transfections

HeLa cells were cultured in DMEM medium (Gibco, Madison, WI, USA) supplemented with 8% FBS (Gibco, Madison, WI, USA) and 1% streptomycin/penicillin (Hyclone, Utah, USA) at 37°C with 5% CO_2_. Transfection with Lipofectamine 2000 (Thermo Fisher Scientific, MA, USA) was performed according to the manufacturer’s instructions. Experiments were performed 24h post transfection.

### Generation of HeLa ICP47 cell line

HeLa cells were transduced with lentivirus containing a C-terminal myc-tagged version of ICP47 (viral inhibitor of TAP transporter) purchased from BrainVTA company (Wuhan, China) and cloned. Expression of ICP47-myc was confirmed by western blot, and inhibition of antigen presentation by HLA I molecules by flow cytometry (**Supplementary Figure 3**). HeLa ICP47 clone 2 was used for the expression of all tagged HLA I molecules used in this study.

### Flow cytometry

**Figure 1:**
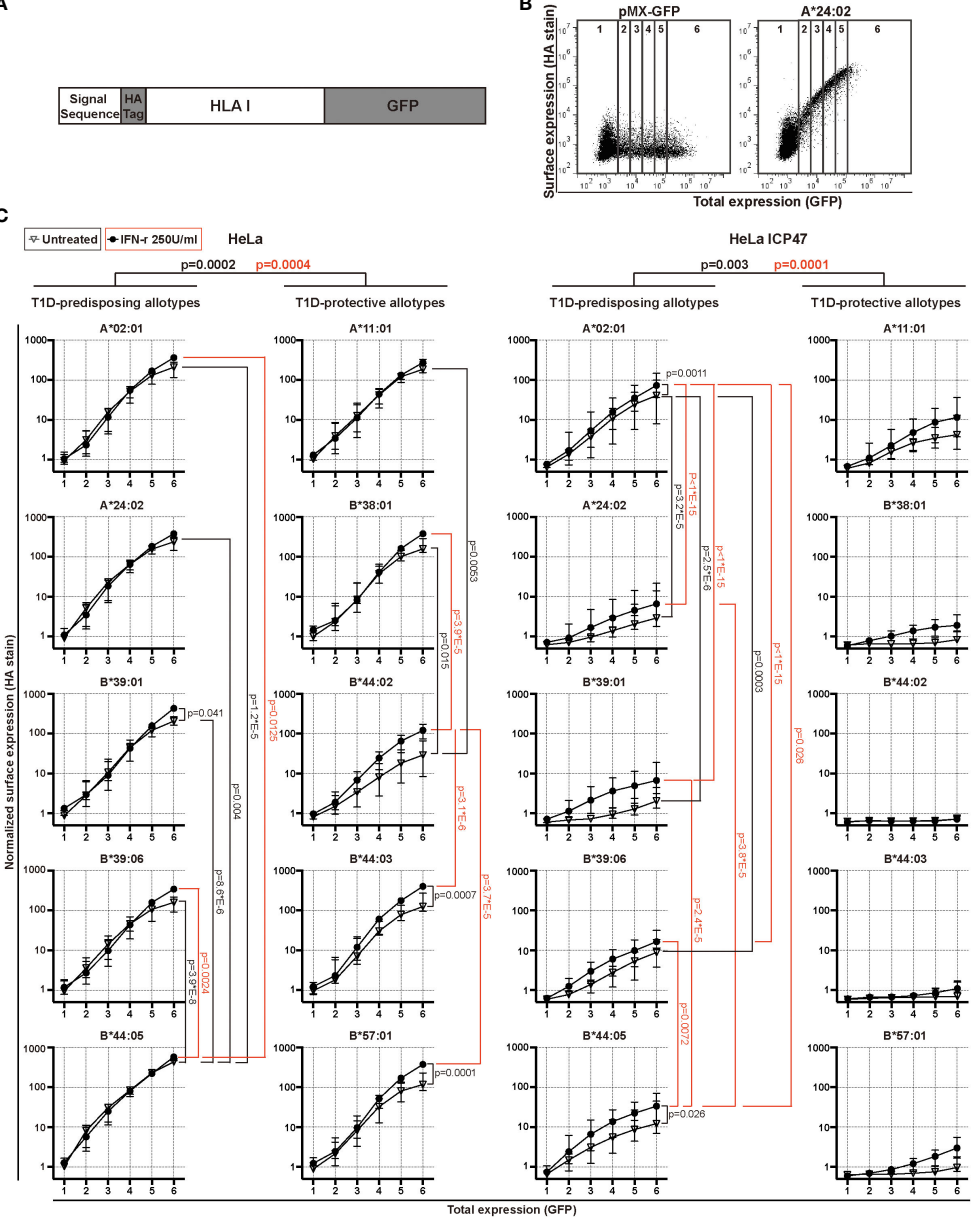
Surface expression of T1D-predisposing and protective HLA I allotypes. **(A)** Schematic representation of the constructs used in the study. **(B, C)** HeLa and HeLa ICP47 cells were transiently transfected with the indicated HLA I heavy chains. An empty vector (pMX-GFP-N1) served as a negative control. Surface expression of HLA I molecules was detected by flow cytometry using a monoclonal anti-HA antibody (clone: 12CA5) and anti-mouse secondary antibody conjugated with Alexa647, while the total expression was detected with a GFP signal. **(B)** Example flow cytometry data (for all representative data, see **Supplementary Figure 4**). The area of the scatter plots displaying GFP (x-axis) versus HA (y-axis) fluorescence was divided into several vertical sectors. Only the sectors containing 100 or more events were used for quantification. **(C)** The quantification of three independent experiments. The mean fluorescence intensity (MFI) of the HA signal was measured and normalized to that in region 1 of A*02:01 in untreated HeLa cells. Data from three independent experiments are represented as median and 95% confidence interval (CI). P values were calculated by two-way ANOVA with Tukey’s multiple comparisons test. Differences between the groups were analyzed by two-way ANOVA and Bonferroni correction. Only significantly different (<0.05) p values are included. GFP, green fluorescent protein; HA, hemagglutinin; IFN-γ, interferon-gamma; HLA, human leukocyte antigen; T1D, type 1 diabetes.

HeLa and HeLa ICP47 cell lines expressing HA-HLA I-GFP molecules were washed twice with PBA buffer [10% BSA (Solarbio, Beijing, China), 0.1% sodium azide (Sigma-Aldrich, MO, USA) in PBS (Solarbio, Beijing, China)], and incubated with monoclonal anti-HA antibody (clone: 12CA5) (a kind gift from Sebastian Springer) and goat anti-mouse secondary antibody conjugated with Alexa647 (Cat.A21235, Invitrogen, CA, USA). Cells were analyzed with CytoFLEX Analyzer Platform (Beckman, CA, USA) and processed with FlowJo software (version 7.6, BD Biosciences, CA, USA). To generate the graphs in **Figure 1C**, we calculated the mean gene expression (GFP) and mean antibody staining intensity (Alexa647) for each of the seven regions, generated by drawing five lines parallel to the Alexa647 axis dividing the GFP vs. Alexa647 scatter plots in six equal consecutive regions corresponding to different GFP fluorescence intensity ranges (**Figure 1B**). We plotted the median values for each region for each HLA I allotype normalized to region 1 of A*02:01 in untreated HeLa cells (**Figure 1C**).

### Inflammation model in HeLa cells

HeLa cells were seeded in a 12-well plate (1.2 X 10^5^ cells/well) and treated with different concentrations of interleukin-1 beta (IL-1β) (Novus biologicals, CO, USA), IFN-γ (Peprotech, IL, USA) and tumor necrosis factor (TNF) (Origene, MD, USA) separately or in combination for 24h. Then, the cells were harvested, stained with monoclonal anti-HLA-A,B,C antibody (clone W6/32) conjugated with FITC (Cat. 311404, Biolegend, CA, USA) and analyzed by flow cytometry. IFN-γ at the concentration of 250 U/ml was chosen to establish inflammatory conditions (**Supplementary Figure 1**).

### mRNA isolation and real time-quantitative PCR

The mRNA was isolated in accordance with the instruction for RNAiso plus kit (Cat. 9108, Takara, Tokyo, Japan). The cDNA reverse transcription was performed with PrimeScript RT reagent kit (Cat. RR047A, Takara, Tokyo, Japan). Real-time qPCR was performed using TB Green Premix Ex Taq II kit (Cat. RR820A, Takara, Tokyo, Japan) according to the specification, and detected by Applied Biosystems StepOne Real-time PCR system (Thermo Fisher scientific, MA, USA). The primers used in this study are listed in **Supplementary Table 1**. The quantification was performed to check the relative mRNA expression based on three independent experiments. Relative mRNA concentrations normalized to the expression of *β-actin* were calculated by ΔΔCt method. β-actin was set as the house-keeping gene.

### Confocal microscopy

HeLa and HeLa ICP47 cells were seeded onto the glass coverslips in 24-well plates (0.6 X 10^5^ cells/well) before transfection. The transfected cells were washed with PBS and fixed with 4% PFA (Beyotime, Shanghai, China) for 30 min. The samples were blocked in 3% BSA for 30 min, then stained with Acti-stain™555 (Cat. PHDH1, Cytoskeleton, CO, USA) and DAPI (Beyotime, Shanghai, China) for 30 min. Samples were mounted with mowiol (Cat. 10852, Sigma-Aldrich, MO, USA) on microscope slides and imaged using Leica SP8 confocal microscope (Leica, Nussloch, Germany). Images were recorded for at least 50 cells per HLA I allotype in three independent experiments. Images were analyzed by ImageJ software (version 1.53, National Institutes of Health, MD, USA).

### Endoglycosidase H digestion

Endoglycosidase H treatment was done according to the manufacturer’s instructions (Novoprotein, Suzhou, China). HeLa cells were lysed, samples (10µg of protein) were denatured at 95°C for 10 minutes and incubated with 2µl EndoH enzyme (500U/ul, Cat. PE017, Novoprotein, Suzhou, China) at 37°C for 3 hours, resolved on 8% SDS-PAGE gel and bands of interest were identified by western blot.

### Computational analysis of peptide binding

Sequences of known T1D auto-antigens (source: *Homo sapiens*), including pre-pro-insulin (NP_000198.1), GAD65 (glutamic acid decarboxylase 65, NP_001127838.1), ZnT8 (zinc transporter 8, NP_776250.2), IAPP (islet amyloid polypeptide, NP_000406.1), IA-2 (insulinoma-associated protein 2, NP_001186692.1), IGRP (islet-specific glucose-6-phosphatase catalytic subunit-related protein, NP_066999.1), CHGA (chromogranin A, AAB53685.1), S100β (S100 calcium-binding protein β, AAH01766.1), ISL1 (islet-1, NP_002193.2), UCN3 (urocortin III, KAI2554905.1), VDBP (vitamin D-binding protein, QXP08766.1), GLIPR1 (GLI pathogenesis-related 1, AAH12510.1), GFAP (glial fibrillary acidic protein, AAH41765.1), KCNK16 (potassium channel subfamily K member 16, AAI11861.1), KIF1A (kinesin-like protein KIF1A, AAI11800.1), PCSK2 (prohormone convertase 1, AAH05815.1), SCG5 (secretogranin V, AAH054349.1) were downloaded from NCBI and used as source to predict peptides (8, 9 and 10-mers) generated by proteasome and delivered to the endoplasmic reticulum by TAP transporter using NetCTLpan1.1 (13). NetMHCpan 4.1 (14, 15) was used to estimate the binding of predicted peptides to all the allotypes. %Rank was used to define binders vs. non-binders. %Rank is a hierarchy of the predicted binding score of a tested peptide concerning the distribution of predicted binding scores of a set of random natural peptides to a respective MHC class I molecule. %Rank is independent of the tendency of certain MHC class I molecules towards lower or higher mean predicted affinities and thus enables inter-specific MHC binding prediction comparisons. Strong binders were defined as the ones with %Rank<0.5, weak binders with 0.5<%Rank<2 and no-binders with %Rank>2 (16). For all the binders (%Rank<2), binding affinity distribution data were plotted and analyzed using GraphPad Prism statistical software (version 9.0, Dotmatics, CA, USA).

### Temperature challenge

HeLa and HeLa ICP47 cells were plated in a 6-well plate (2.5 X 10^5^ cells/well), transfected and grown for 24h. Cells were lysed, and lysates were incubated under different temperatures (4°C, 37°C and 50°C) for 10 min and returned instantly to 4°C. Supernatants were subjected to immunoprecipitation with W6/32 antibody that recognizes peptide-bound forms of HLA I molecules (a kind gift of Jacques Neefjes) prebound to protein A/G agarose beads (HB180824, Yeasen, Shanghai, China) at 4°C overnight. The peptide-bound forms of HLA I molecules were dissociated from the beads by heating at 95°C for 10 minutes and analyzed by western blot using anti-GFP monoclonal antibody (Cat. 66002-1-Ig, Proteintech, USA).

### Western blot

Cells were lysed in a buffer containing 0.5% CHAPS (Sigma-Aldrich, MO, USA) and a Complete protease inhibitor cocktail without EDTA (Roche, Basel, Switzerland) in PBS. Proteins were then resolved on SDS-PAGE gel and transferred to the PVDF membrane. The membrane was blocked with 5% milk in PBST [PBS supplemented with 0.1% Tween-20 (Sigma-Aldrich, MO, USA)] at room temperature for 1h. Membranes were incubated with primary antibodies [anti-GFP monoclonal antibody (Cat.66002-1-Ig, Proteintech, IL, USA), anti-myc antibody (Cat.2278, Cell Signaling Technology, MA, USA), anti-HLA ABC antibody (Cat.ab70328, Abcam, Cambridge, United Kingdom), anti-tapasin antibody (Cat.PA5-42731, Invitrogen, CA, USA), anti-GAPDH antibody (Cat.6004-1-Ig, Proteintech, IL, USA)] at 4°C overnight, washed with PBST and incubated with HRP-conjugated secondary anti-mouse antibody (Cat. 405306, Biolegend, CA, USA) or HRP-conjugated secondary anti-rabbit antibody (Cat. 406401, Biolegend, CA, USA) at room temperature for 1h. The membranes were visualized with the ECL kit (Cat. 32109, Thermo Fisher Scientific, MA, USA) on the Imager 5200 (Tanon, Shanghai, China).

### Frustration analysis

An online available dataset containing frustration analysis data of HLA I allotypes in the complex with 10 different peptides was downloaded (17). Frustration data for the first 180 amino acids (constituting peptide-binding groove) of 10 HLA I allotypes (A*02:01, A*24:02, B*39:01, B*39:06, B*44:05, A*11:01, B*38:01, B*44:02, B*44:03, B*57:01) were extracted from the dataset by frustratometer2 tool (https://github.com/gonzaparra/frustratometer2) (18). The heatmap and boxplots, which represent frustration index values, were generated for each amino acid residue by GraphPad Prism (version 9.0, Dotmatics, CA, USA).

### Mutational analysis

A*02:01 (PDB: 5HHN), A*24:02 (PDB: 7JYV), B*39:01 (PDB: 4O2E), A*11:01 (PDB: 6JOZ) and B*57:01 (PDB: 2RFX) were input into DynaMut [https://biosig.lab.uq.edu.au/dynamut/] (19) and change in the change in Gibbs free energy (ΔΔG), a metric to predict whether a point mutation is favorable in terms of protein stability, was computed.

### Statistical analysis

GraphPad Prism (version 9.0, Dotmatics, CA, USA) was used to analyze the data. Quantitative variables were represented as median and error, and categorical variables were described using absolute and relative frequencies. Box and whisker plots indicate the median with range (min-max). The Shapiro–Wilk test was used to verify the normal distribution. Kruskal-Wallis and Dunn’s multiple comparison test was used for non-parametric test. For comparisons between the two groups, an unpaired Mann-Whitney test was performed. To compare three or more data sets, a one-way ANOVA with Tukey’s multiple comparison analysis was used to determine differences. If the Brown-Forsythe ANOVA test indicated an unequal variance in the standard deviation of experimental groups being compared, a Welch’s ANOVA with Dunnett’s T3 multiple comparisons test was used. Two-way ANOVA with the Geisser–Greenhouse correction for repeated measures was used to monitor parameters over fluorescence or temperature range, followed by Tukey’s multiple comparisons test. The Fisher’s exact test was used for frequency data, followed by Bonferroni correction for multiple testing. The *p*-value less than 0.05 was considered to be statistically significant.

## Results

### T1D-predisposing allotypes have higher cell surface levels than protective allotypes

HLA I molecules present peptides at the cell surface to cytotoxic CD8^+^ T cells. In T1D, inflammation promotes HLA I hyperexpression at the cell surface of pancreatic β cells, possibly contributing to the activation of CD8^+^ T cells and β cell destruction (4). This suggests that HLA I surface expression is closely related to the occurrence of T1D (20). Thus, we first determined the surface levels of T1D-predisposing, A*02:01 (5, 8), A*24:02 (5, 6, 8, 9), B*39:01 (21, 22), B*39:06 (5, 7, 8), B*44:05 (5) and protective, A*11:01 (5, 6, 8, 9), B*38:01 (6, 8), B*44:02 (5, 8), B*44:03 (5, 7, 8), B*57:01 (5, 7, 8), HLA I allotypes under normal and inflammatory conditions present in T1D. We expressed each HLA I allotype with N-terminal HA [located after the signal sequence as done previously (23–25)] and C-terminal GFP tag (**Figure 1A**). HA stain indicated HLA I surface expression, whilst GFP provided total expression levels (**Figure 1B**). IFN-γ, IL-1β and TNF, the main inflammatory cytokines in T1D, were titrated to establish inflammatory conditions defined as increased HLA I surface levels (**Supplementary Figure 1**). Treatment with only IFN-γ at 250U/ml was enough to induce surface HLA class I levels and was chosen for follow-up experiments. B*44:05 allotype showed higher surface levels than the remaining T1D-predisposing allotypes. B*44:02 had lower surface levels than A*11:01 and B*38:01. Overall, T1D-predisposing allotypes had higher surface levels than protective allotypes in wild-type HeLa cells. Surface levels were increased upon IFN-γ treatment concurrent with the increased expression of chaperones in the HLA I antigen presentation pathway (**Supplementary Figure 2**). Still, B*44:05 had higher levels than the A*02:01 and B*39:06, while B*44:02 had lower surface expression than most other T1D-protective allotypes studied (**Figure 1C**). Cumulatively, T1D-predisposing HLA I allotypes had higher surface levels than T1D-protective allotypes.

In T1D, an inverse correlation was found between CD8^+^ T cell response and peptide affinity to HLA I (26), and higher frequencies of CD8^+^ T cells recognizing very low-affinity peptides presented by HLA I than those recognizing the peptides with intermediate affinities (27), indicating that presentation of low-affinity peptides by HLA I might contribute to autoimmunity. To create the conditions that allow addressing presentation of low-affinity (sub-optimal) peptides by T1D-predisposing and protective allotypes, peptide transport by TAP was inhibited by the Herpes simplex virus-encoded protein, ICP47 (28) (**Supplementary Figure 3**). Upon limited peptide supply, HLA I surface levels were reduced, that was more pronounced for the T1D-protective subtypes. A*02:01 had higher surface levels than most other T1D-predisposing allotypes. T1D-protective allotypes showed similar plasma membrane expression under peptide-limiting conditions. IFN-γ treatment increased the expression of A*02:01 and B*44:05 but not of the protective allotypes (**Figure 1C**). Cumulatively, T1D-predisposing HLA I allotypes had higher surface levels than T1D-protective allotypes under peptide-limiting conditions. These data suggest that T1D-predisposing allotypes can proceed to the cell surface more easily than protective HLA-B allotypes.

### T1D-predisposing and protective HLA I allotypes have similar maturation status

**Figure 2:**
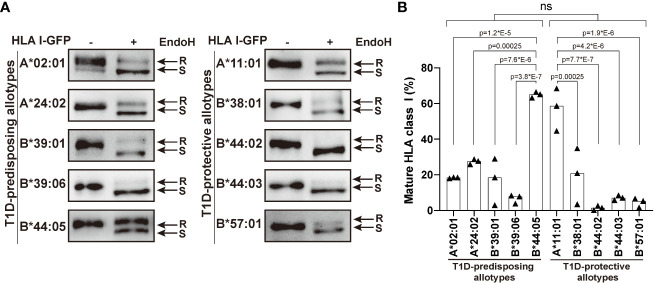
Maturation of T1D-predisposing and protective HLA I molecules. HeLa cells were transiently transfected with the indicated HLA I heavy chains fused with GFP. Half of the lysates were either treated with Endoglycosidase H (EndoH) or mock-treated before separation by 8% SDS-PAGE, transfer and detection with anti-GFP (1E10H7) antibody. **(A)** Representative western blots for each allotype. R, EndoH-resistant form; S, EndoH-sensitive form. **(B)** Quantification of three independent experiments. Percentage of mature HLA I was calculated as a ratio of the EndoH-resistant form to the total (sum of EndoH-resistant and EndoH-sensitive forms). Data are represented by scatter dot plot with median. Statistical significance was determined by one-way ANOVA and Tukey’s multiple comparisons test. Differences between the groups were analyzed by Welch’s t-test. P values <0.05 were considered significant and are included in the figure; ns, non-significant.

Higher surface levels may result from an increased maturation rate. Therefore, we next asked whether T1D-predisposing and protective allotypes differ in their rate of exit to the cell surface. All secretory glycoproteins, including HLA I molecules, undergo transfer of initial N-glycan(s) in the ER and their subsequent modifications when they pass through medial Golgi, resulting in the formation of mature glycans. Thus, mannose-rich oligosaccharides indicate ER forms, while complex N-linked oligosaccharides indicate Golgi and post-Golgi forms of secretory glycoproteins that can be differentiated with the enzyme endoglycosidase H (EndoH), which only cleaves mannose-rich glycans from the glycoproteins (so-called EndoH-sensitive forms). EndoH-sensitive proteins are believed to remain inside the cell, while EndoH-resistant proteins proceed to the cell surface. We performed EndoH digestion to detect the extent of HLA I intracellular transport, as previously described (11, 29). Most HLA I allotypes were predominately in EndoH-sensitive form. B*44:05 showed a significantly higher proportion of mature forms than other T1D-predisposing allotypes, however there were no differences between T1D-predisposing and protective allotypes (**Figure 2**). As the differences in maturation do not correspond to the cell surface levels, maturation may not be the main factor affecting differences in surface expression between T1D-predisposing and protective allotypes.

### T1D-predisposing allotypes are localized more at the cell periphery than T1D-protective allotypes

**Figure 3:**
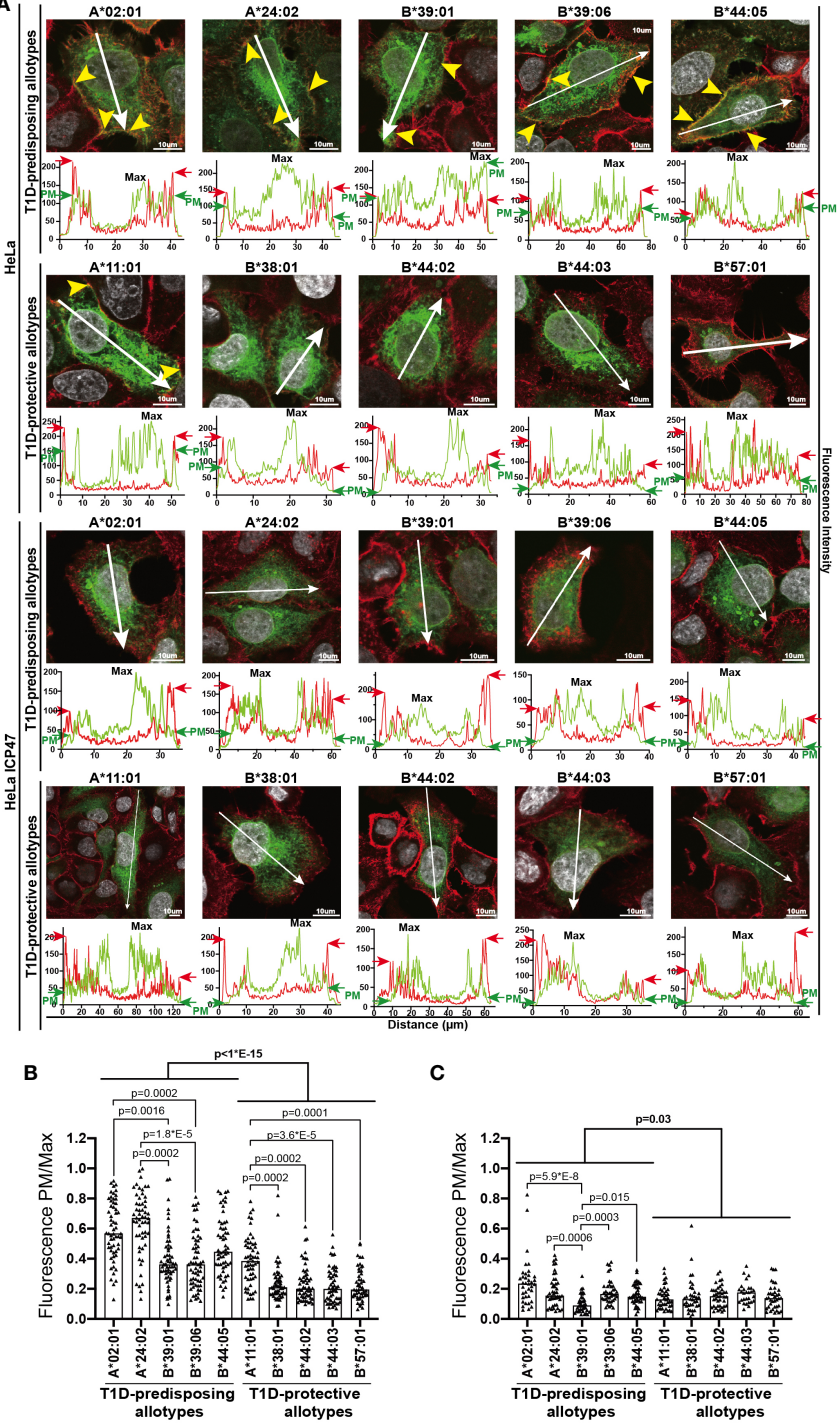
Intracellular localization of T1D-predisposing and protective HLA I allotypes. HeLa and HeLa ICP47 cells were transfected with HLA I–GFP (green), stained with actin marker, phalloidin (red) and DNA marker DAPI to stain nucleus (white), and imaged by confocal microscopy. **(A)** Representative merged images are shown (for the field of cells and single stains, see **Supplementary Figure 5**). The co-distribution of HLA I with phalloidin is marked with yellow arrows. Fluorescence intensity histograms over indicated lines are shown below each image. Green arrows (GFP) indicate peaks originating from the HLA I-GFP signal at the edge of the cell [PM, plasma membrane, further confirmed by cooccurrence with red arrows (phalloidin)], while “Max” shows the maximum expression of HLA I-GFP within the cell. HLA I-GFP distribution was analyzed and represented as a ratio of PM/Max for HeLa **(B)** and HeLa ICP47-myc **(C)** cells. More than 50 cells with similar GFP signal (corresponding to the GFP intensity in regions 2-5 in **Figure 1B**) were assessed for each allotype, scale bar 10 μm. Data from three independent experiments are represented by a scatter dot plot with a median. Statistical difference was analyzed by Kruskal-Wallis and Dunn’s multiple comparisons test. Differences between the groups were analyzed by Mann-Whitney test. Only significantly different (<0.05) p values are included.

To understand the reason for the differential surface levels of T1D-predisposing and protective allotypes, we next studied their cellular distribution by immunofluorescence microscopy adapted from Pan and colleagues (30) through assessing the co-distribution of HLA I-GFP with actin marker, phalloidin. T1D-predisposing allotypes and A*11:01 showed uniform distribution throughout the cell. In contrast, protective HLA-B allotypes were primarily localized near the nucleus and at a lower density in the cell periphery (**Figure 3A**), further confirmed by quantifying HLA I fluorescence intensity at the cell edges versus maximum intensity (Fluorescence PM/Max, **Figure 3B**). Interestingly, HLA-A allotypes, A*02:01 and A*24:02, showed higher PM/Max than the remaining T1D-predisposing allotypes. At the same time, A*11:01 had higher PM/Max than the remaining protective allotypes. Overall, T1D-predisposing allotypes showed higher surface localization than the protective allotypes. In the peptide-limiting environment, all allotypes had changed intracellular distribution as compared to peptide-proficient conditions, low levels at the edge of the cell as compared with phalloidin stain, and robust intracellular localization near the nucleus (**Figure 3**). Still, T1D-predisposing allotypes localized to the higher degree at the plasma membrane than T1D-protective allotypes. T1D-predisposing allotypes are more likely to localize at the cell periphery than protective allotypes. These data, taken together with our flow cytometry results (**Figure 1**), suggest that T1D-predisposing allotypes undergo a less stringent quality control that regulates HLA I export to the cell surface and have the potential to present peptides more readily with less stringency than T1D-protective allotypes.

### T1D-predisposing allotypes have more peptide-bound forms than protective allotypes

**Figure 4:**
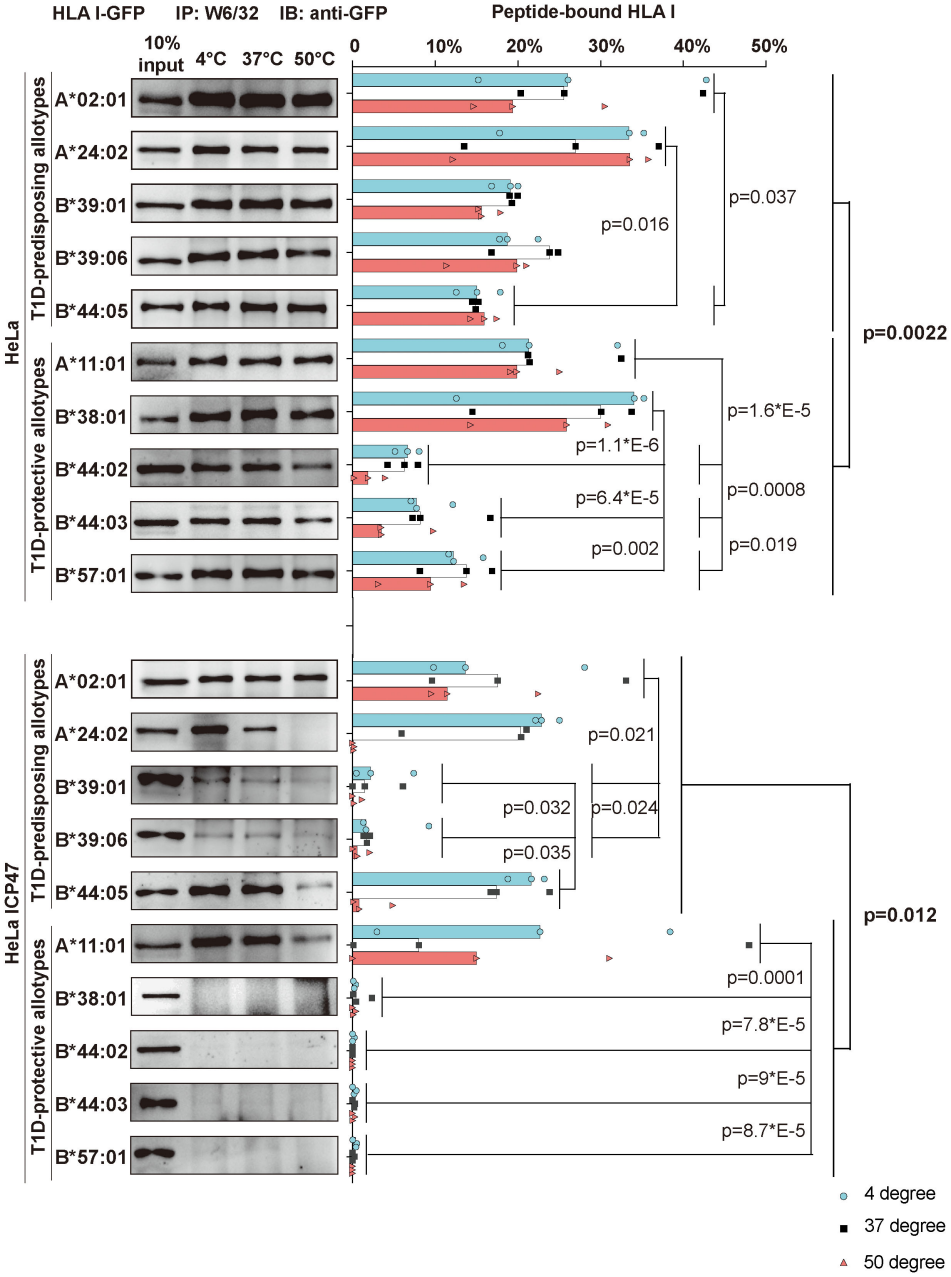
Peptide binding and thermal stability of T1D-predisposing and protective HLA I allotypes. HeLa and HeLa ICP47 cells were transiently transfected with the indicated HLA I heavy chains fused to GFP. Cell lysates were incubated under different temperatures (4°C, 37°C, and 50°C) for 10 min and immediately after, being placed at 4°C. Peptide-bound forms of HLA I were isolated using W6/32 antibody and visualized by western blot with anti-GFP antibody. The percentage of peptide-bound forms was calculated as W6/32-reactive HLA I allotype as the percentage of the total of this allotype. The representative membrane for each allotype from three independent experiments is shown. Data from three independent experiments are represented by scatter dot plot with median. P values were calculated by two-way ANOVA with Tukey’s multiple comparisons test. Differences between the groups were analyzed by two-way ANOVA and Bonferroni correction. Only significantly different (<0.05) p values are included. IP, immunoprecipitation; IB, immunoblot.

HLA I molecules expressed on the plasma membrane in cells with non-functional TAP are known to present low-affinity (suboptimal) peptides (31, 32). Thus, the surface expression of T1D-predisposing allotypes on HeLa ICP47 (**Figure 1**) cells may suggest that T1D-predisposing allotypes bind and present suboptimal peptides. Therefore, we next compared peptide binding between T1D-predisposing and protective HLA I allotypes. HeLa cells expressing HLA I-GFP constructs were lysed. Lysates were heated to induce the dissociation of unstable peptide-HLA I complexes. The remaining peptide-bound HLA I molecules were isolated through immunoprecipitation with W6/32 antibody, which recognizes peptide and β_2_m-bound HLA I in lysates (33) as previously described (34). In HeLa cells, A*02:01 and A*24:02 had more peptide-bound forms than B*44:05, while A*11:01 and B*38:01 more peptide-bound forms than the other T1D-protective allotypes. Overall, T1D-predisposing allotypes had a higher proportion of peptide-bound forms than T1D-protective allotypes, suggesting that they bind peptides more efficiently. (**Figure 4**, upper part). Upon limited peptide supply, T1D-predisposing allotypes and HLA-A*11:01 still formed complexes with peptides, but protective HLA-B allotypes did not (**Figure 4**, lower part), indicating that T1D-predisposing allotypes and A*11:01 may bind sub-optimal peptides. In addition, A*02:01 and A*24:02 showed more peptide-bound forms than B*39:01 and B*39:06, and A*11:01 than the remaining protective allotypes. Also, in Hela ICP47 cell line, T1D-predisposing allotypes, had a higher proportion of peptide-bound forms than T1D-protective allotypes (**Figure 4**). Taken together, T1D-predisposing allotypes may be more promiscuous in peptide selection (35) and bind peptides more easily than protective allotypes, which corresponds to their higher surface levels, suggesting that peptide binding and presentation might explain the differential association of HLA I molecules with T1D.

### T1D-protective allotypes bind a significant proportion of 10-mers

**Figure 5:**
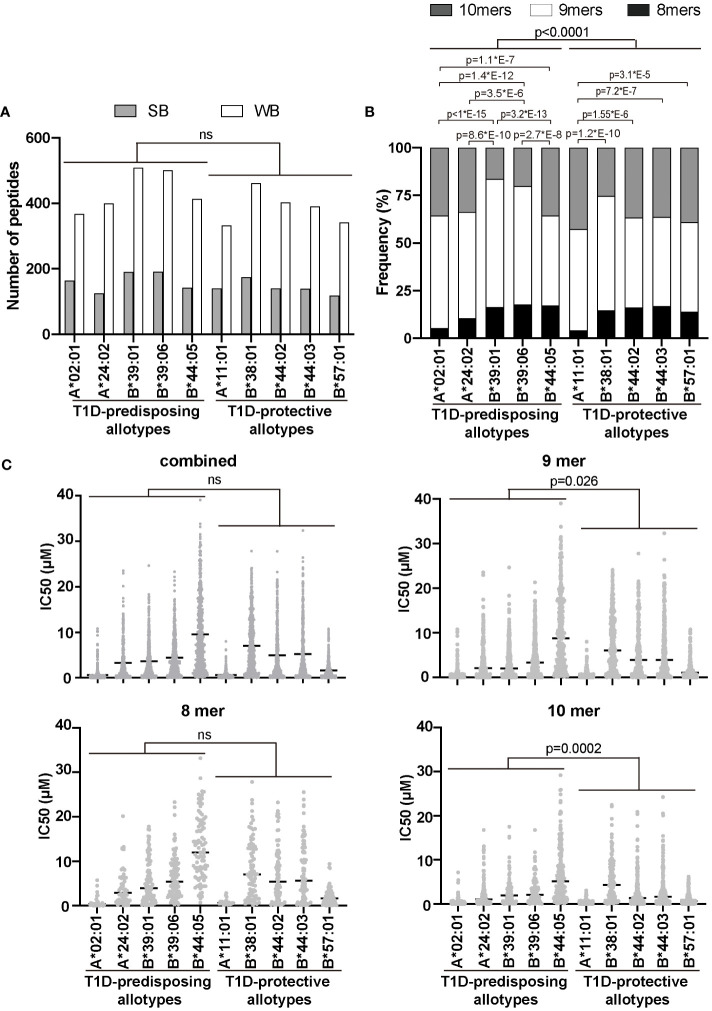
Characteristics of peptidomes of T1D-predisposing and protective HLA I allotypes. Sequences of peptides (8, 9 and 10-mers) derived from T1D auto-antigens produced by proteasome and delivered to the ER by TAP transporter were predicted using NetCTLpan1.1 (13) (for all the predicted peptides see **Supplementary Table 2**). Peptide binding to HLA I molecules was predicted using NetMHCpan4.1 and %Rank (14–16). **(A)** Number of strong binders (SB, %Rank<0.5) and weak binders (WB, 0.5<%Rank<2). Number of non-binders (NB, %Rank>2) is not shown. Statistical significance was analyzed using Fisher’s exact test and Bonferroni correction. **(B)** Frequency of binders (%Rank<2) with different lengths. Statistical significance was analyzed using Fisher’s exact test and Bonferroni correction. **(C)** The binding affinity of the predicted peptidome is represented by IC_50_ by scatter dot plot with median. Statistical differences between T1D-predisposing and protective allotypes were determined using Man-Whitney test. For multiple comparisons see **Supplementary Table 5**. ns, non-significant.

Given that T1D-predisposing and protective allotypes differ in the amount and stability of peptide-bond forms, we next explored whether T1D predisposition is affected by the quality of peptides they bind. We first selected 17 T1D auto-antigens, including pre-pro-insulin, and used their sequences to predict peptides produced by the proteasome and delivered to the ER by the TAP transporter (13). A total of 22,242 identified peptides were predicted whether they can bind to HLA I allotypes, as done previously (32). A total of 3287 peptides were predicted to bind to at least one of the tested HLA I allotypes, with 1268 predicted to bind to more than one HLA class I molecule (**Supplementary Tables 2**–**4**). Among identified possible binders, 74 were previously reported to be presented by HLA I molecules, mostly by A*02:01, in T1D (36–39) (**Supplementary Tables 2**, **3**), which strengthens our *in sillio* approach. The numbers of strong binders (SB) and weak binders (WB) did not differ between allotypes, and we did not see differences between T1D-predisposing and protective groups (**Figure 5A**). Most allotypes preferentially bound 9-mers (**Supplementary Figure 6**). However, 9-mers were predicted to bind with the higher IC_50_ than 10-mers to most of the studied allotypes (**Supplementary Figure 7**). T1D-protective allotypes bound a higher ratio of 10-mers than predisposing allotypes (**Figure 5B**; **Supplementary Figure 6C**). In addition, 10-mers bound to T1D-protective allotypes had lower IC_50_ than the 10-mers bound to T1D-predisposing allotypes, indicating higher affinity (**Figure 5C**). These results suggest that T1D-protective allotypes can bind 10-mers better than T1D-predisposing allotypes and may thus select peptides with 10 amino acids more often than T1D-predisposing allotypes.

### T1D-predisposing and protective allotypes differ in sequence and energetic profile of the F pocket

**Figure 6:**
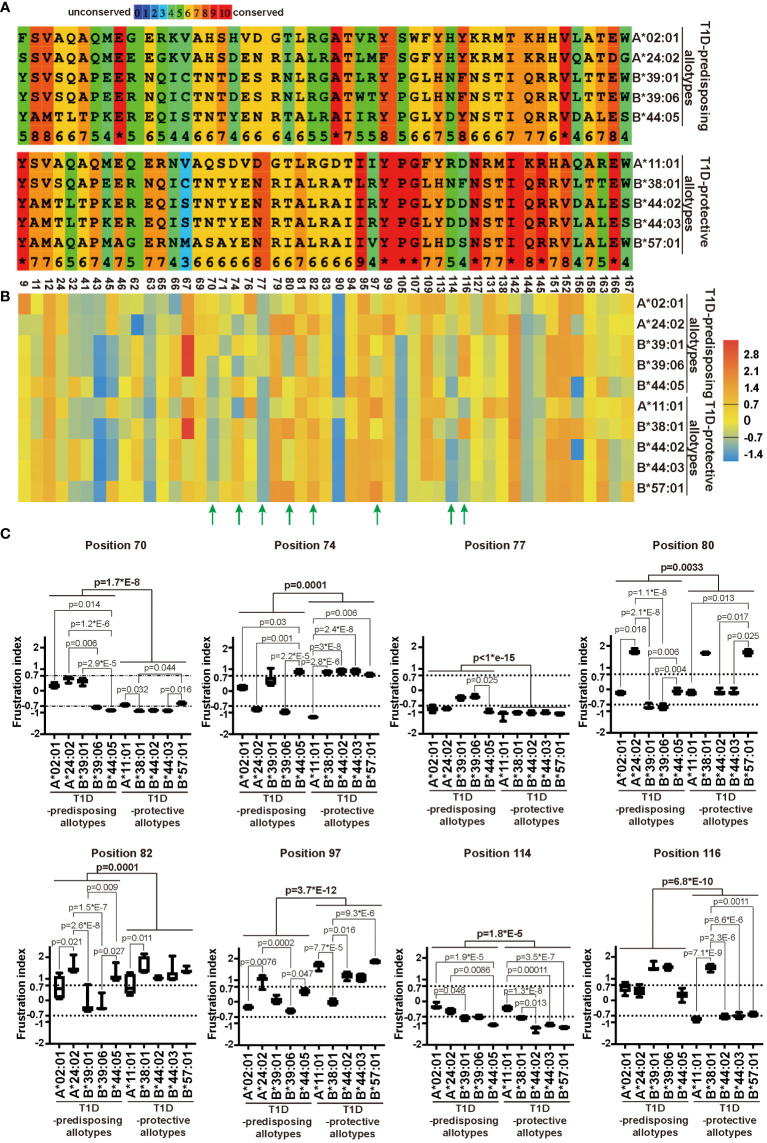
Sequence variation and energetic optimization of residues in the peptide-binding groove in T1D-predisposing and protective HLA I allotypes. **(A)** HLA I sequences were aligned using Praline (developed in the Centre for Integrative Bioinformatics Vrije Universiteit Amsterdam). The most polymorphic residues in the peptide-binding groove of HLA I allotypes included in this study were selected and represented. For the entire sequences of peptide-binding grooves, see **Supplementary Figure 8**. Amino acid conservation is in the color-coded and numeric representation. Amino acids are represented by a single letter code; specifically, A, alanine; C, cysteine; D, aspartic acid; E, glutamic acid; G, glycine; F, phenylalanine; H, histidine; I, isoleucine; K, lysine; L, leucine; M, methionine; N, asparagine; P, proline; Q, glutamine; R, arginine; S, serine; T, threonine; V, valine; W, tryptophan, Y, tyrosine. The numbers at the bottom indicate the residue number. **(B, C)** Single Residue Frustration Indices (SRFI) of the polymorphic residues in peptide-binding grooves were downloaded from the online dataset (40). The median SRFI was calculated for each polymorphic residue from 100 peptide-HLA I models (10 models per allotype) of T1D-predisposing and protective allotypes and shown by heat map **(B, C)**. The SRFIs of residues with a substantial difference between T1D-predisposing and protective allotypes are marked with green arrows **(B)** and illustrated by box plots with whiskers (min-max) **(C)**. A frustration index less than -0.7 is defined as high frustration, while one more than 0.7 as minimal frustration. The number between -0.7 and 0.7 stands for neutral frustration. Statistical difference was analyzed by Kruskal-Wallis and Dunn’s multiple comparisons test. Differences between the groups were analyzed by Mann-Whitney test. Only significantly different (<0.05) p values are included.

The sequence of a protein determines the folding and, consequently, the function (40). As we observed differences in localization and function between T1D-predisposing and protective allotypes, we next assessed how these differences are governed by genetic polymorphism and structural variations between allotypes. We compared the amino acid sequences of the peptide-binding groove (positions 1-180) of all the allotypes. We observed that most differences were located within regions containing residues 62-83 and residues 90-116. T1D-protective allotypes share more conserved regions than predisposing allotypes, except position 116 (**Supplementary Figure 8**; **Figure 6A**).

The polymorphic residues may have different interactions with the neighboring residues that might affect their structural stability, as observed before using the concept of local frustration corresponding to the energetic profile of a given residue in the structure (40). Minimally frustrated residues are believed to have optimized the energetic contacts with neighboring residues and are conformationally more stable. On the other hand, highly frustrated residues are postulated to not have optimal interactions with the neighboring amino acids, therefore may be conformationally unstable and may introduce destabilizing effects into the peptide binding pockets (40). The frustration indices for all the polymorphic amino acids within peptide-binding grooves for T1D-predisposing and protective HLA I allotypes were taken from the online dataset (17), and mean frustration indices were represented as a heatmap. T1D-predisposing and protective allotypes showed differences in frustration profiles for residues 70, 74, 77, 80, 82, 97, 114 and 116, most of which are known to comprise the F pocket in the HLA I peptide binding groove (**Figure 6B**) (10). Next, we detailed frustration indices for these residues, including frustration data for each HLA I allotype based on models with ten different peptides. Positions 97 and 116 showed the most prominent variations between T1D-predisposing and protective allotypes (**Figure 6C**). Position 97 was minimally frustrated in T1D-protective allotypes, except B*38:01, and neutrally frustrated in predisposing allotypes, except A*24:02. On the other hand, position 116 showed high frustration in T1D-protective allotypes, except B*38:01, and minimal frustration in predisposing allotypes, suggesting that residue 116 may exert stabilizing effects on the F pocket in T1D-predisposing allotypes. Besides, positions 77 and 114 were highly frustrated in T1D-protective allotypes while showing overall lower frustration in predisposing allotypes. Residues 77 and 116 in HLA I interact with peptide side chains, and residue 114 modulates F pocket specificity (10). In T1D-predisposing allotypes, position 116 harbors Tyr or Phe, bulky hydrophobic amino acids known to reduce flexibility and increase the local conformation stability. On the other hand, Asp or Ser present at position 116 in most T1D-protective allotypes may disturb the conformation of the F pocket when not stabilized by interaction with residue 114 (11, 34). Higher frustration of residue 116 in T1D-protective allotypes may indicate that this position has not optimized interactions with the neighboring residues that could negatively affect HLA I conformational stability of the peptide-binding groove, dictate HLA I surface expression and consequently govern the T1D predisposition.

### Position 116 impacts HLA I predisposition to T1D

**Figure 7:**
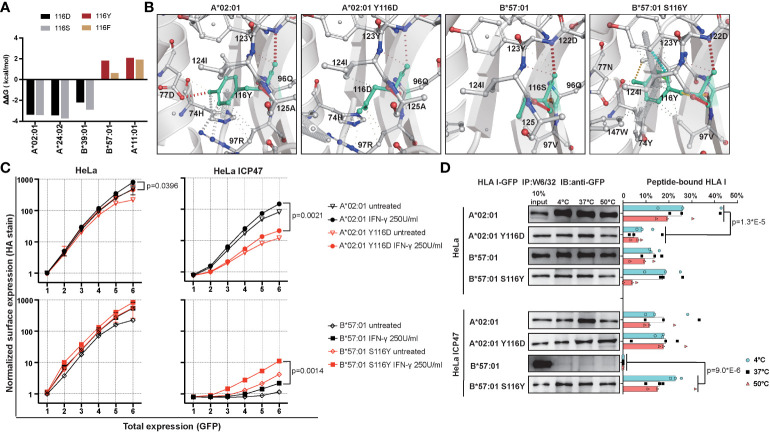
Characterization of mutants with changed F pocket architecture. **(A)** Stability prediction by DynaMut web server (19) based on PDB files, A*02:01 (PDB: 5HHN), A*24:02 (PDB: 7JYV), B*39:01 (PDB: 4O2E), A*11:01 (PDB: 6JOZ) and B*57:01 (PDB: 2RFX). **(B)** F pocket interactions in A*02:01 WT and Y116D mutant, B*57:01 WT and S116Y mutant. **(C)** Surface expression of WT and mutated A*02:01 and B*57:01 in HeLa and HeLa ICP47 cell lines under normal and inflammatory conditions, example flow cytometry data (for all representative data, see **Supplementary Figure 9**). Data from two independent experiments are represented by median and 95% CI (confidence interval). **(D)** Thermal stability of A*02:01 WT, B*57:01 WT and corresponding mutants in HeLa and HeLa ICP47 cell lines. WT, wild-type. Data from three independent experiments are represented by scatter dot plot with median. P values in **(C, D)** were calculated by two-way ANOVA with Tukey’s multiple comparisons test. Only significantly different (<0.05) p values are included.

Our above observation suggested the position 116 located in the F pocket, defines HLA I peptide binding ability and surface expression. To test our hypothesis that position 116 in F pocket is responsible for differences between T1D-predisposing and protective HLA class I allotypes, we decided to exchange residue at position 116 to see how this may affect the peptide binding groove. We decided to exchange Tyr/Phe to Asp/Ser in T1D-predisposing allotypes, and Asp/Ser to Tyr/Phe in T1D-protective allotypes. Mutating Asp to Tyr in B*44:02 yields B*44:05, so we excluded B44 allotypes from the analysis. We employed DynaMut [https://biosig.lab.uq.edu.au/dynamut/ (19)], which allows to predict the effect of mutation on protein stability based on its crystal structure. As crystal structures of B*38:01 and B*39:06 are unavailable, we decided to input A*02:01, A*24:02, B*39:01, A*11:01 and B*57:01 into DynaMut. Exchanging position 116 from Tyr/Phe to Asp/Ser in T1D-predisposing allotypes had a destabilizing effect as indicated by negative value of Gibbs free energy (ΔΔG<0), while mutating it from Asp/Ser to Tyr/Phe increased the stability (ΔΔG>0) of HLA I molecules (**Figure 7A**). Mutations in A*02:01 and A*24:02 had similar destabilizing effects, so we selected the first one from four mutants, specifically A*02:01 Y116D. Considering T1D-protective allotypes, as properties of A*11:01 partially resemble that of T1D-predisposing allotypes, we selected B*57:01. In B*57:01, mutation S116Y had a stronger stabilizing effect than mutation S116F. In summary, A*02:01 Y116D and B*57:01 S116Y mutants were selected to be tested. Exchanging Ser to Tyr in B*57:01 resulted in gain in the interactions of position 116 with residues 74, 77, 97, 123 and 147. Specifically, Tyr116 in B*57:01 showed an additional hydrogen bond and ionic interactions with Asn77, ionic and hydrophobic interactions with Val97, hydrophobic interactions with Trp147 and Tyr74, and additional ionic interactions with Tyr123, suggesting a more stable F pocket. At the same time, mutating Tyr to Asp led to a reduction in interactions with residues 74, 77 and 97 (**Figure 7B**). A*02:01 Y116D lost hydrogen bond with Asp77, cation-π interaction with Arg97, and π-π interaction with His74, suggesting a less stable F pocket. In agreement with the prediction, A*02:01 Y116D mutant showed lower surface stain than the corresponding WT molecule, in HeLa and HeLa ICP47 cells under inflammatory conditions, while B*57:01 S116Y had higher levels than that of the WT counterpart in IFN-γ-treated HeLa ICP47 cells (**Figure 7C**). Compared to the WT molecule, A*02:01 Y116D showed a reduced amount of peptide-bound forms in HeLa cells. In contrast, B*57:01 S116Y increased capacity to bind peptides in HeLa ICP47 (**Figure 7D**). Thus, the amino acid differences at the key position 116 in A*02:01 seemed sufficient to make the T1D-predisposing allotype behaves like a protective allotype and vice versa. This indicates that the residue 116 may serve as a key molecular signature to differentiate between T1D-predisposing and protective allotypes.

## Discussion

HLA I antigen presentation is implicated in the pathogenesis of T1D. HLA I molecules are hyper-expressed on pancreatic β cells (4), and CD8^+^ T cells recognize self-antigens presented by HLA I molecules (2). Besides, certain *HLA I* alleles genetically predispose to T1D (6, 8). However, it remains unknown why some *HLA I* alleles increase the risk of T1D, whereas others do not, and the underlying molecular mechanism has not been elucidated. Here, we performed a comprehensive comparison between T1D-predisposing and protective allotypes using various biochemical, cell biology and *in silico* approaches to understand what structural and functional features could make HLA I allotype predisposing to T1D. We found that T1D-predisposing allotypes had higher surface expression and post-ER localization than protective allotypes. Besides, T1D-predisposing allotypes bound more suboptimal peptides. Moreover, T1D-predisposing allotypes were predicted to bind a higher proportion of 9-mers and to bind 9-mer peptides with higher affinity than T1D-protective allotypes. T1D-predisposing and protective allotypes displayed different energetic patterns in the peptide-binding groove, with T1D-predisposing allotypes being less frustrated at residues that comprise pocket F, including position 116. Mutating position 116 in A*02:01 and B*57:01 yielded mutants with properties resembling that of T1D-protective and T1D-predisposing allotypes, respectively. Overall, our research found that the F pocket residue 116 may be crucial for T1D predisposition.

### Peptide binding and presentation are the main factors governing T1D predisposition

We found that 9 amino acids are the preferred length of peptides binding to both T1D-predisposing and protective allotypes (**Figure 5B**), which is in agreement with previous mass spectrometry analyses (41, 42). By comparing predicted binding affinities of peptidomes of different lengths, we observed that 10-mers showed higher binding affinity than 9-mers, and 9-mers showed higher binding affinity than 8-mers to most of the analyzed HLA class I allotypes (**Supplementary Figure 7**). It has been reported that B*39:01 bound 9-mer SHVAVENAL with higher predicted affinity than 8-mer HVAVENAL, and the resulting complex had higher thermal stability (43). Similarly, A*24:02 bound 10-mer, IYFSPIRVTF, with higher affinity than 9-mer, YFSPIRVTF, and the resulting complex had higher thermal stability (44). Interestingly, protective allotypes were predicted to select more 10-mers than predisposing allotypes and bind 10-mers with higher affinity. Predisposing allotypes selected more 9-mers, even though 9-mers showed overall lower affinity than 10-mers. Taken together, T1D-protective allotypes prefer to select higher affinity peptides than predisposing allotypes. This suggests that predisposing allotypes are less dependent on high-affinity peptides than protective ones.

Besides, T1D-protective HLA-B allotypes had fewer peptide-bound forms in HeLa and almost none in HeLa ICP47 cell lines, indicating that they may be restricted to binding high-affinity peptides. On the contrary, T1D-predisposing allotypes were more likely to bind peptides, even in the presence of ICP47. As a reduction in TAP activity results in a significant decrease in optimal peptides for HLA I molecules (31, 32), our data indicate that T1D-predisposing allotypes might bind suboptimal peptides (29).

We found that T1D-predisposing allotypes showed higher surface levels than protective allotypes in HeLa and HeLa ICP47 cells, suggesting that T1D-predisposing allotypes escaped cellular quality control (45) and presented suboptimal peptides at the cell surface. Although inflammation increased the cellular levels of antigen processing and presentation machinery (**Supplementary Figure 2**) (46), some neopeptides might be produced during the inflammation (47), and we observed increased surface levels of T1D-predisposing allotypes under inflammatory conditions, T1D-protective HLA-B allotypes remained inside the cell, indicating that T1D-predisposing allotypes are more likely than protective allotypes to present peptides to CD8^+^ T cells.

HLA class I undergoes peptide optimization with the help of tapasin within the peptide-loading complex. A*02:01, A*24:02, B*39:01, B*39:06, B*44:05 and A*11:01 display low, B*38:01 intermediate, and B*44:02, B*44:03 and B*57:01 high tapasin dependence for folding, peptide optimization and surface expression (48, 49). These suggest that T1D-protective HLA-B allomorphs undergo tapasin-mediated peptide optimization, while T1D-predisposing allotypes may proceed to the cell surface binding suboptimal peptides. Indeed, diabetogenic auto-antigens presented by HLA class I molecules were found to be of very low-affinity (27). Thus, tapasin editing may exclude autoreactive ligands from HLA class I allotypes.

Surprisingly, in both groups, HLA-A allotypes showed more peptide-bound forms and higher surface levels in the HeLa ICP47 cell line than HLA-B allotypes. As A*02:01, A*24:02 and A*11:01 can bind signal peptides (50), they can form peptide-HLA I complexes even in the absence or inhibition of TAP transporter (51). Still, T1D-predisposing A*02:01 and A*24:02 had more peptide-bound forms than T1D-protective A*11:01, suggesting their lower restriction in peptide selection or better peptide optimization. This could be due to the fact that A*02:01 and A*24:02 have higher preference to undergo TAPBPR-mediated peptide editing than other HLA I allotypes (52). Overall, T1D-predisposing allotypes seem promiscuous and can bind and present suboptimal peptides. On the other hand, T1D-protective allotypes seem fastidious and bind peptidomes of higher affinity. This may result in a broader range of CD8^+^ T cell clones reactive to T1D-predisposing than protective HLA I allotypes (35). Previous studies have suggested that promiscuous HLA I may be prone to cause autoimmunity (53).

T1D is accompanied by ER stress and unfolded protein response (UPR) (4). UPR hinders HLA I antigen presentation by inhibiting the generation of cytosol-derived, but not ER-derived, peptides (54). As ICP47 blocks peptide transport to the ER, it resembles peptide-limiting conditions present under UPR which comprise of signal peptides and other antigens generated in the ER or delivered to the ER in TAP-independent manner (55, 56). Under such peptide-limited conditions, T1D-predisposing allotypes still bound and presented suboptimal peptides, while protective allotypes did not. Presentation of such suboptimal (low-affinity) peptides at the cell surface might activate autoreactive CD8^+^ T cells, as such CD8^+^ T cells are not eliminated during negative selection. Inflammation in T1D might further enhance surface levels of T1D-predisposing allotypes and augment the aberrant activation of auto-reactive cytotoxic CD8^+^ T cells due to increased avidity (multiplicity of peptide-HLA I-TCR interactions) (2).

### Residue 116 in F pocket in HLA I structure correlates with T1D predisposition

T1D-predisposing allotypes are more conserved than protective allotypes at residue 116 in the F pocket, harboring bulky Phe or Tyr that have optimized interactions within HLA I structure to bind peptides with lower conformational flexibility, as we demonstrated by minimal frustration. In contrast, T1D-protective allotypes showed highly frustrated positions 116 and 114. Residue 116 strongly affects peptide binding due to the interaction with a side chain of peptide C terminus (10, 32, 34). Residue 114 interacts with residue 116 and defines pocket F specificity (10). In T1D-protective B*44 and B*57:01, the negatively charged residue 114 (Asp) causes steric hindrances in the binding groove, which might hinder peptide binding. On the contrary, positively charged residues in HLA-A allotypes (Arg and His) might enhance HLA peptide binding ability, and increase the preference to bind TAPBPR (52, 57). When Asp at position 114 is accompanied by Asp at position 116 in T1D-protective B*44 allotypes, the F pocket is more flexible and less conformationally stable, as we and others observed previously (34, 58). Similarly, Asp114 accompanied by Ser116 in B*57:01 may result in a more flexible peptide-binding groove. Lastly, Asn114 may also contribute to the increased flexibility of the HLA I structure depending on the surrounding residues. In B*44:05, Tyr116 reduced conformational flexibility and stabilized the F pocket (34). The optimized interactions and lower conformational flexibility within the F pocket of T1D-predisposing allotypes might define the set of peptides that this allotype can bind, induce more peptide-bound forms, more stable peptide-HLA I complexes and higher surface expression. Indeed, B*57:01 S116Y had more stable peptide binding groove, was more permissive in binding suboptimal peptides, and had higher cell surface levels. In contrast, the A*02:01 Y116D mutant had less peptide-bound forms and lower cell surface levels than its wild-type counterpart.

Intriguingly, A*11:01, the T1D-protective allotype, showed a similar percentage of the peptide-bound form and surface expression as T1D-predisposing HLA-B allotypes. This might be due to a strong interaction between negatively charged Asp116 and positively charged Arg114. This was only found in A*11:01 but not in any other T1D-protective allotypes, which could explain the fact that A*11:01 allotype had the lowest frustration within the T1D-protective allotypes at position 114 and higher than T1D-predisposing HLA-A allotypes at position 116, resulting in less peptide-bound forms and lower surface expression than T1D-predisposing HLA-A allotypes. We propose that the polymorphism at positions 114 and 116 might explain the association of HLA I with T1D.

It must be noted that T1D-predisposing B*39:01 and B*39:06, and T1D-protective B*38:01 all have Asn at 114 and Phe116 and similar frustration of these positions. Yet, these HLA-B allotypes show differential peptide-binding and surface levels. B*39 allotypes and B*38:01 differ by six amino acids at positions 74, 77, and 80-83 within the F pocket. T1D-protective B*38:01 showed higher frustration of residue 77 than the B*39 allotypes. Residue 77 in T1D-predisposing B*39:01 (Ser77) forms hydrogen bond with peptide position 9, and van der Waals contacts with positions 8 and 9 (43). As residue 77 in HLA I molecules interacts with the peptide C terminus and defines F pocket specificity (10), it may additionally modulate the conformational stability of HLA I. Thus, F pocket architecture may govern T1D predisposition, with the main contribution governed by residues 114 and 116 and secondary effects by residue 77.

### Other factors that could promote T1D via HLA I antigen presentation

HLA class II allotypes were suggested to contribute to the initiation of immune response and generation of T1D auto-antibodies by presenting antigens to the auto-reactive CD4^+^ T cells, whereas HLA class I to contribute to the progression of β cell damage by presenting peptides to auto-reactive CD8^+^ cytotoxic T cells (59). Besides the polymorphism in HLA I genes, other factors might affect HLA I antigen presentation and, consequently, the activation of CD8^+^ T cells in T1D. Enteroviruses were proposed to trigger T1D in HLA-prone individuals by inducing inflammation, including IFN-γ, that augments HLA I surface expression on pancreatic β cells and infiltration of immune cells into pancreatic islets (4). Besides, two genotypes from eight polymorphisms of the IFN-γ locus in intron 1, which increase IFN-γ production, were associated with susceptibility to T1D (57). Increased IFN-γ levels might inhibit the expression of anti-inflammatory cytokines such as IL-10, breaking normal immune tolerance (60). Furthermore, IFN-γ can induce the cellular levels of antigen processing and presentation machinery (1), including TAP1, tapasin and TAPBPR, which may augment HLA I antigen presentation and explain the increased surface levels of HLA I molecules we expressed in HeLa cells (**Supplementary Figure 2**). In addition, the polymorphism of TAP2 (TAP2*0101) and ERAP1 (rs30187 and rs26618) might enhance the risk of T1D (61, 62). Although TAP2*0101 polymorphism was found to have a small effect on peptide transportation *in vitro*, the study did not address the effect of inflammation present in T1D (63). ERAP1 SNP rs30187 positively correlated with T1D in the British population (64). However, this association was not observed in the study within an Italian child population (62). The ERAP1 SNP rs30187-encoded amino acid substitution K528R reduced the capability of ERAP1 to trim peptides (65), which could result in a higher proportion of suboptimal peptides for HLA I molecules. ERAP1 SNP rs26618, located in the third position in codon 276, was hypothesized to affect the structure and function of ERAP1 in autoimmune diseases, although this hypothesis lacks experimental data (66). Overall, environmental factors that induce inflammation in T1D might contribute to the higher frequency of suboptimal peptides and augmented surface presence of T1D-associated HLA I molecules. Under such conditions, T1D-predisposing allotypes that might bind and present such suboptimal peptides might contribute to the pathogenesis of T1D.

Most of the mass spectrometry analyses of peptides binding to human HLA class I was done on allotypes isolated from non-pancreatic cells. To our knowledge, there is only analysis of the ligandome of HLA class I molecules from ECN90 pancreatic cell line unstimulated or stimulated with IFN-γ and IFN-γ/TNF/IL-1β. ECN90 cell line expresses A*02:01, A*03:01, B*40:01, B*49:01, C*03:04, C*07:01. Around 43% of eluted peptides were shared between unstimulated and stimulated conditions, and 63% of peptides were either only presented or enriched in the stimulated cells. A*02:01 had the highest number of predicted peptides (n=32), with other allotypes having from 3 to 20 predicted peptides (47). More work is needed to compare β cell ligandomes of T1D-predisposing and protective allotypes under basal and stimulated conditions and assess whether certain peptides are presented by T1D-predisposing allotypes but not by the protective ones.

## Conclusions

We demonstrated that T1D-predisposing allotypes had a higher capacity to bind and present suboptimal peptides under inflammatory conditions resembling the ones present in T1D. This was dictated by the gene polymorphism within the F pocket, especially residue 116. Energetic profiles of residues 114 and 116 in T1D-predisposing allotypes allowed optimized interactions with the peptide and surrounding residues and contributed to a more stable conformation. Targeting position 116 may reduce stability and decrease surface expression of T1D-predisposing HLA I allotypes, which might result in reduced activation of CD8^+^ T cells in T1D. Our findings need to be confirmed in the human pancreatic β cells. In conclusion, what we found here is not only limited to T1D therapy but also gives a new insight into the study and treatment of other autoimmune diseases.

## Data availability statement

The original contributions presented in the study are included in the article/**Supplementary Material**. Further inquiries can be directed to the corresponding author.

## Author contributions

XR: Data curation, Formal analysis, Investigation, Visualization, Writing – original draft, Writing – review & editing. AA: Formal analysis, Investigation, Visualization, Writing – review & editing. MJ: Formal analysis, Investigation, Visualization, Writing – review & editing. MG: Conceptualization, Funding acquisition, Investigation, Project administration, Resources, Supervision, Writing – original draft, Writing – review & editing.
