# Supplementary material for: Differences in F pocket impact on HLA I genetic associations with autoimmune diabetes
Source: Front Immunol. 2024 Mar 25;15:1342335. doi: 10.3389/fimmu.2024.1342335 (PMC11003304; doi:10.3389/fimmu.2024.1342335)
Supplement: Supplementary file 1 [file Image_1.pdf]

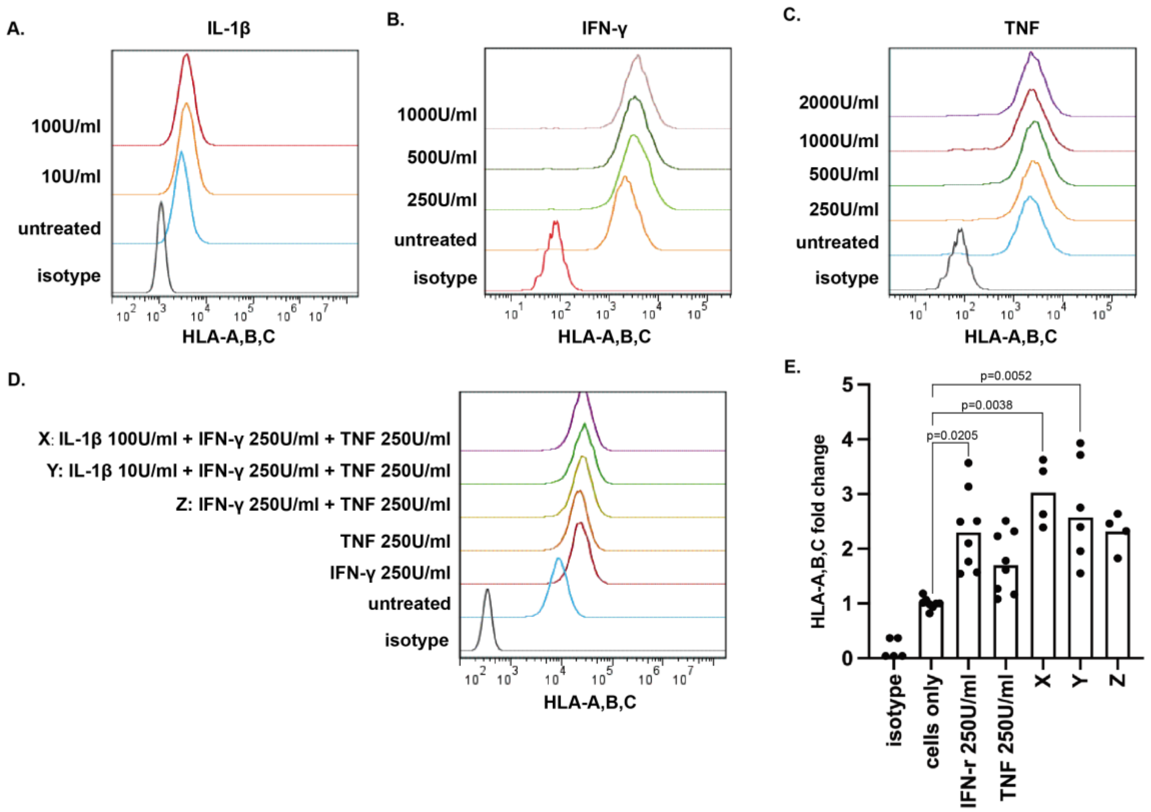

Supplementary Figure 1 corresponding to Figure 1C. Establishing inflammatory conditions in HeLa cells.

HeLa cells were seeded in a 12-well plate (1.2 X 10^5 cells/well) and treated with different concentrations of IL-1β, IFN-γ and TNF separately or in combination for 24h. Surface expression of HLA I was detected by flow cytometry using monoclonal anti-HLA-A,B,C antibody (clone W6/32) conjugated with FITC. **(A-D).** Representative histograms of single cytokine treatment **(A-C)** or combined treatment of "IL-1β 100U/ml + IFN-γ 250U/ml + TNF 250U/ml" (marked as X in the figure), "IL-1β 10U/ml + IFN-γ 250U/ml + TNF 250U/ml" (marked as Y in the figure) and "IFN-γ 250U/ml + TNF 250U/ml" (marked as Z in the figure) **(D)**. **(E)**. Quantification from three independent experiments. Statistical difference was analyzed by Kruskal-Wallis test and Dunn's multiple comparisons test. IFN-γ at the concentration of 250 U/ml was selected to establish inflammatory conditions. HLA – human leukocyte antigen; IFN-γ, interferon-gamma; IL-1β, interleukin 1 beta; TNF; tumor necrosis factor.

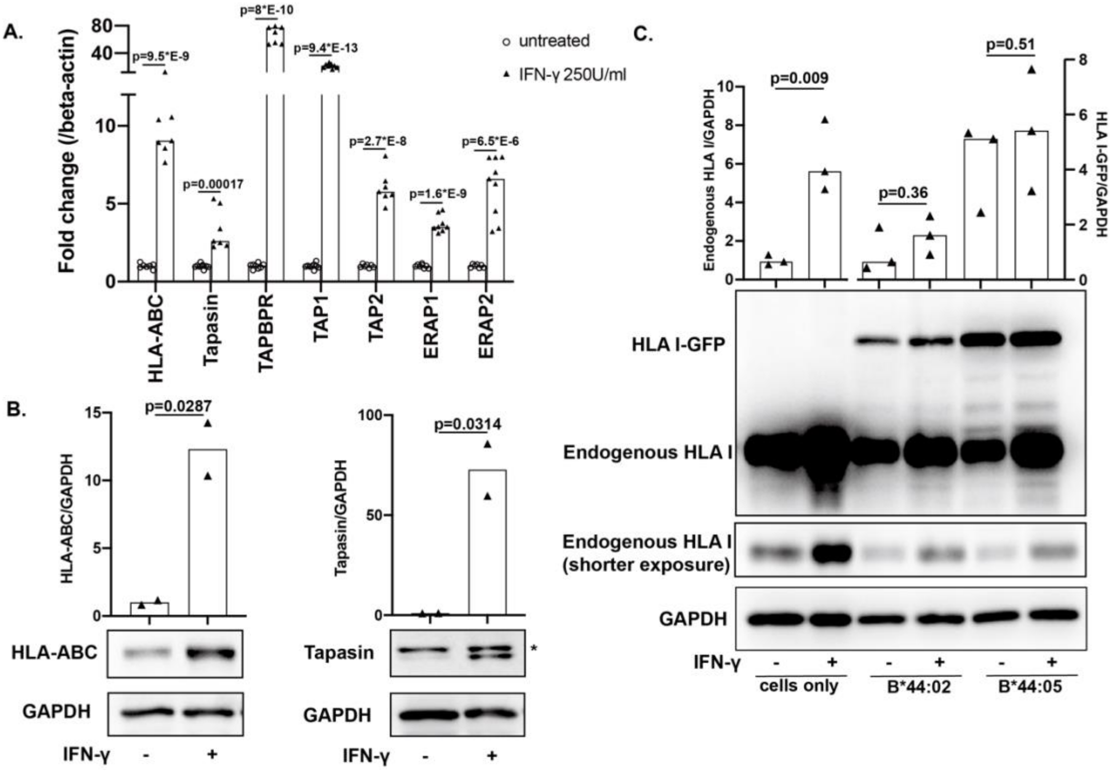

Supplementary Figure 2 corresponding to Figure 1C. IFN-γ increases expression of endogenous HLA I molecules and other components of HLA I antigen presentation pathway.

(A). The mRNA expression of HLA-ABC, tapasin, TAPBPR, TAP1, TAP2, ERAP1 and ERAP2 under IFN-γ treatment in HeLa cells analyzed by qPCR. **(B).** Protein expression of endogenous HLA I and tapasin under IFN-γ treatment in HeLa cells. *non-specific band. **(C).** Effect of IFN-γ on transfected HLA I-GFP and endogenous HLA I protein expression in HeLa cells. Statistical significance was analyzed by unpaired t-test for normally distributed data and Welch's t-test for data that were not distributed normally.

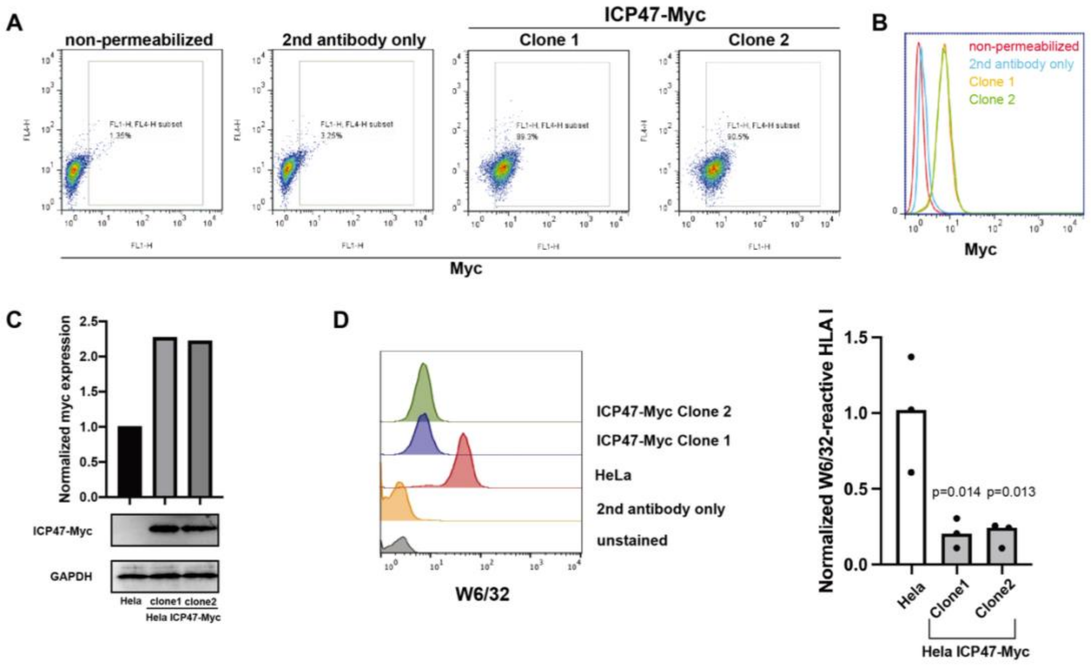

Supplementary Figure 3 corresponding to Figure 1C. Generation of HeLa ICP47-myc stable cell line.

HeLa cells were stably lentivirally transduced with a C-terminal myc-tagged version of ICP47 (viral inhibitor of TAP transporter) and cloned. Stable expression of ICP47-myc was confirmed by flow cytometry **(A-B)** and western-blot **(C)** using an anti-myc monoclonal antibody (clone 71D10) and anti-mouse secondary antibody conjugated with HRP. Quantification: Normalized myc expression was calculated as a ratio of the ICP47-myc band to the GAPDH band. **(D).** Inhibition of antigen presentation by HLA I molecules was confirmed by flow cytometry with anti-HLA-A,B,C monoclonal antibody (clone W6/32) conjugated with FITC. Quantification: Normalized W6/32–reactive HLA I was calculated as a ratio of the expression in ICP47-myc- expressing cells to WT HeLa cells. Data are represented by scatter dot plot with median. Statistical difference was analyzed using one-way ANOVA and Tukey's multiple comparisons test. Clone2 was selected for all the experiments included in this manuscript. HRP, horseradish peroxidase; TAP, transported associated with antigen processing, WT, wild-type.

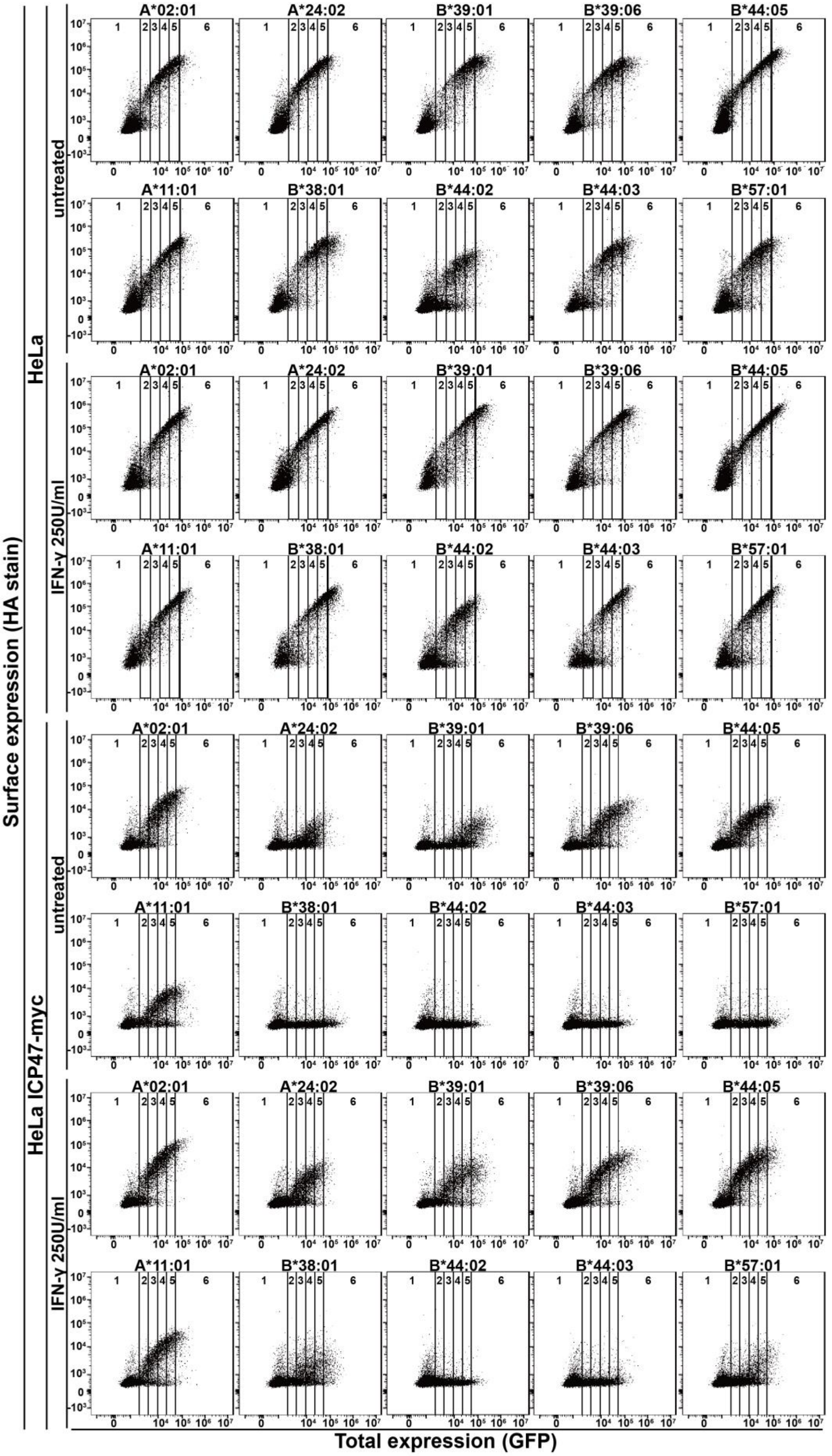

Surface expression (HA stain)
Total expression (GFP)
HeLa
HeLa ICP47-myc
untreated
IFN-γ 250U/ml
A*02:01
A*24:02
B*39:01
B*39:06
B*44:05
A*11:01
B*38:01
B*44:02
B*44:03
B*57:01
1
2
3
4
5
6

Supplementary Figure 4 corresponding to Figure 1C. The surface expression of T1D-predisposing and protective HLA I allotypes – all representative flow cytometry data.

HeLa and HeLa ICP47-myc cells expressing the indicated HLA I heavy chains (HA-HLA I- GFP) were stained with anti-HA antibody (clone:12CA5) to analyze HLA I allotype expression at the cell surface. The total expression is shown by the GFP signal. The area of the scatter plots indicating GFP (x-axis) versus HA (y-axis) fluorescence was divided into several vertical sectors. GFP, green fluorescent protein; HA, hemagglutinin; T1D, type 1 diabetes.

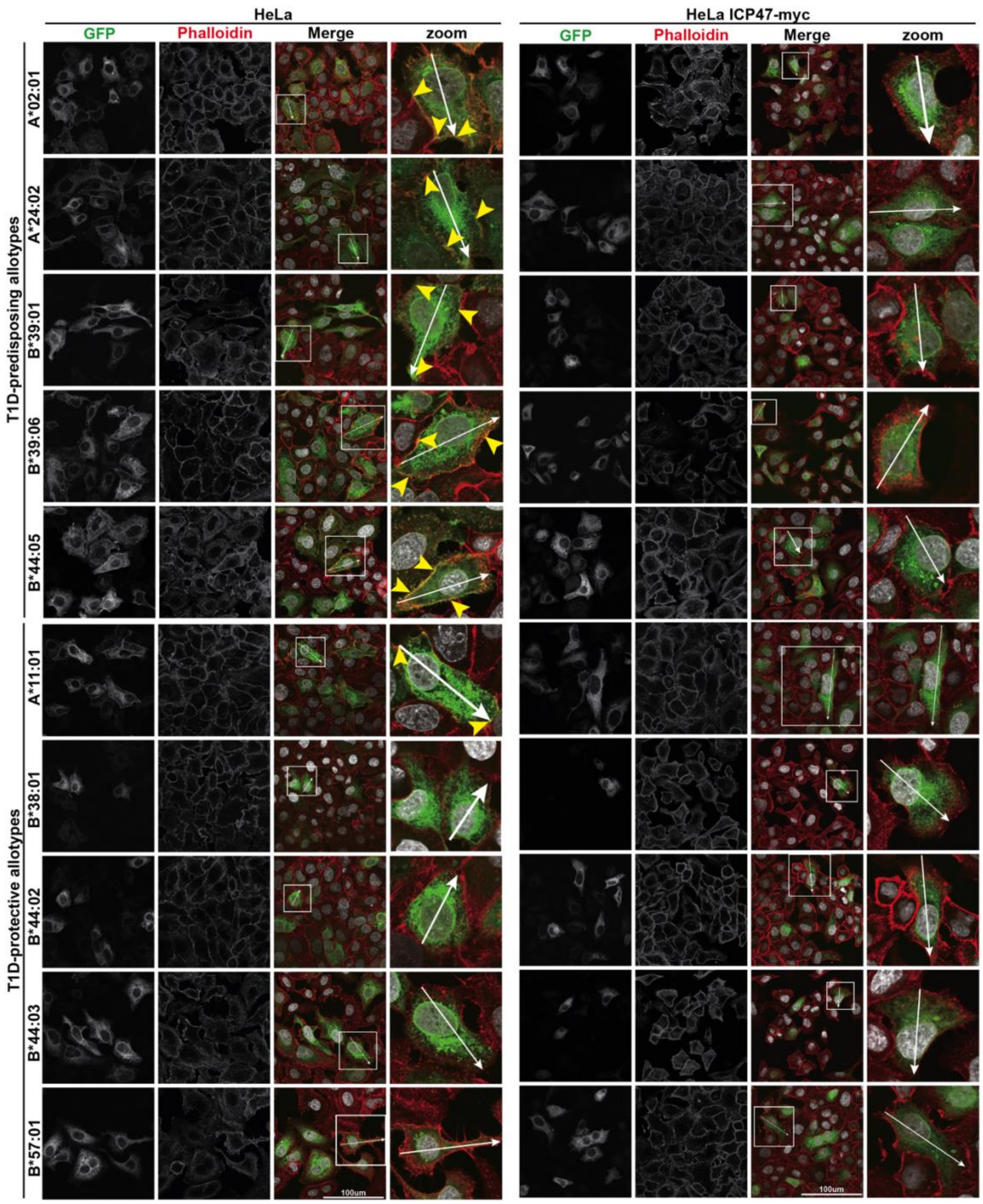

Supplementary Figure 5 corresponding to Figure 3. Intracellular localization of T1D-predisposing and protective HLA I allotypes – field of cells and single stains.

HeLa and HeLa ICP47-myc cells expressing the indicated HLA I molecules with GFP tag (green) were stained with phalloidin (red) and imaged by confocal microscopy. Representative single stains, merged and zoomed images are shown. The co-distribution of HLA I with phalloidin is marked with yellow arrows; scale bar 100 μm.

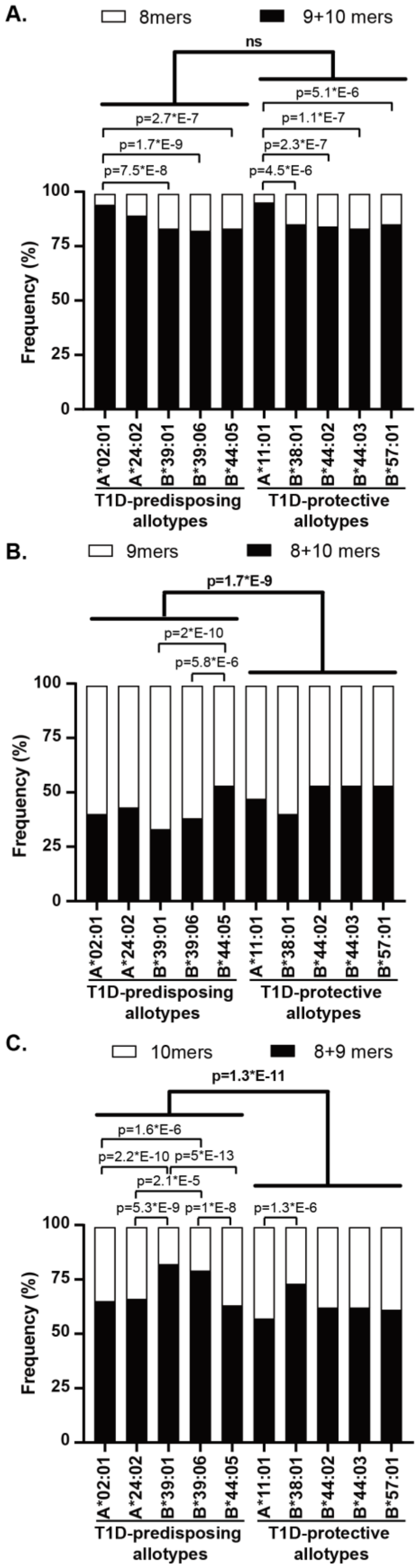

Supplementary Figure 6 corresponding to Figure 5. T1D-predisposing allotypes bind more 9-mers than protective allotypes. Analysis of the length distribution in binders (%Rank<2) of T1D-predisposing and protective allotypes. **(A).** Frequency of 8-mers versus 9-mers and 10-mers. **(B).** Frequency of 9-mers versus 8-mers and 10-mers. **(C).** Frequency of 10-mers versus 8-mers and 9-mers. Statistical significance was analyzed using Fisher’s exact test and Bonferroni correction.

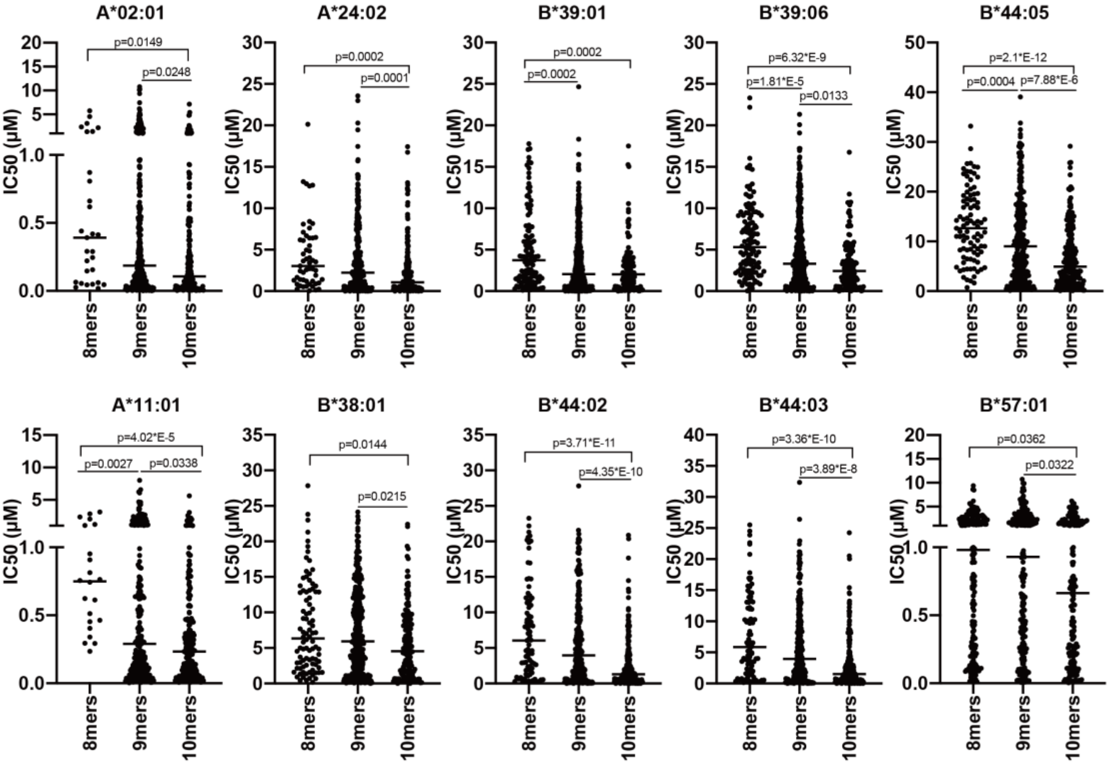

Supplementary Figure 7 corresponding to Figure 5C. Binding affinity predictions for 8-mers, 9-mers and 10-mers. The binding affinity of the predicted peptidome is represented by IC_{50} by scatter dot plot with median. Data are represented as scatter dot plot. Statistical differences between peptides of different length (8-mers, 9-mers and 10-mers) for each HLA I allele were determined using Kruskal-Wallis with multiple Dunn's multiple comparisons test.

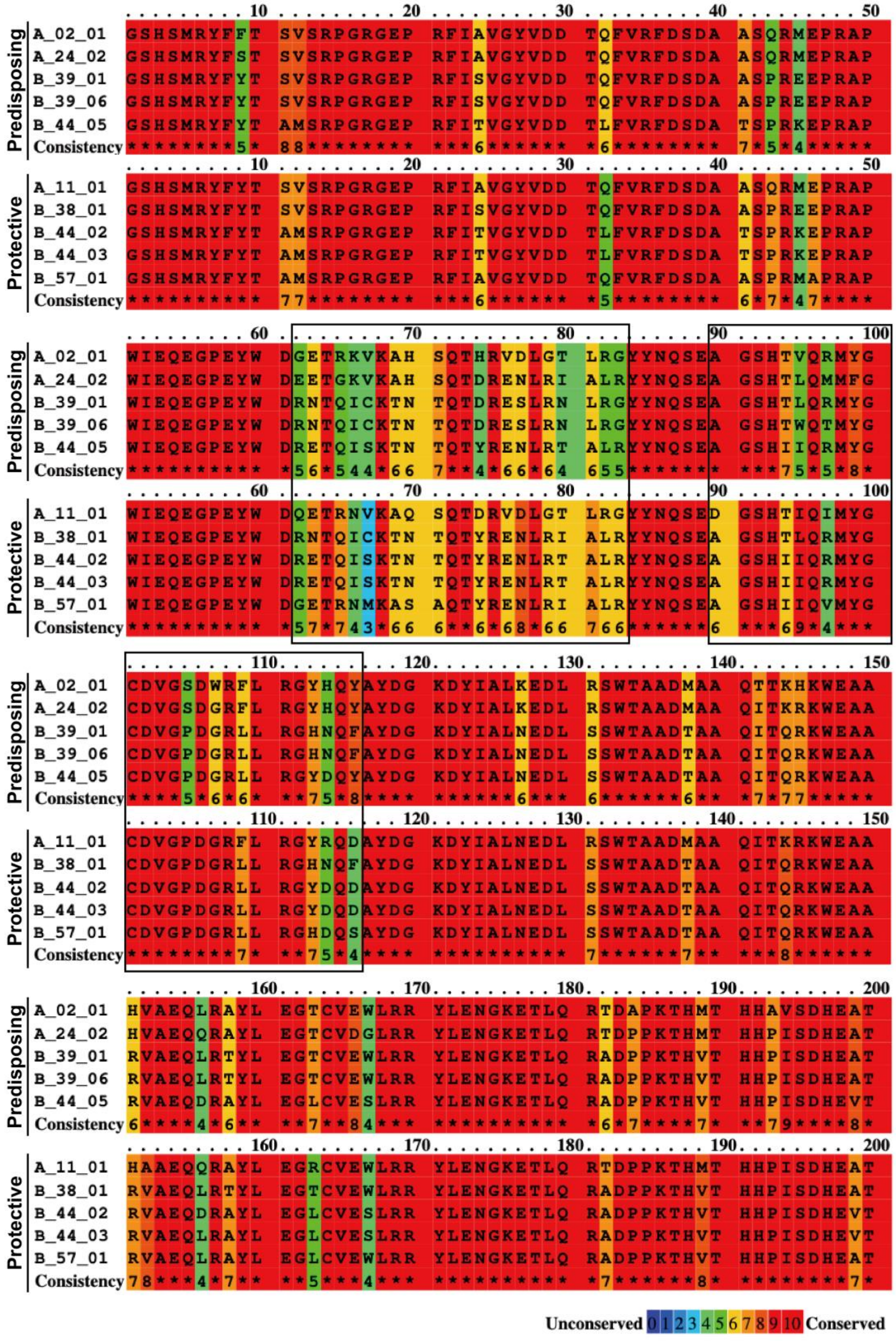

Supplementary Figure 8 corresponding to Figure 6. Amino acid sequence alignment of T1D-predisposing and protective HLA I allotypes.

HLA I sequences of the peptide-binding groove were aligned using Praline (developed in the Centre for Integrative Bioinformatics Vrije Universiteit Amsterdam). Amino acid conservation is in the color-coded and numeric representation. Amino acids are represented by a single letter code; specifically, A, alanine; C, cysteine; D, aspartic acid; E, glutamic acid; G, glycine; F, phenylalanine; H, histidine; I, isoleucine; K, lysine; L, leucine; M, methionine; N, asparagine; P, proline; Q, glutamine; R, arginine; S, serine; T, threonine; V, valine; W, tryptophan, Y, tyrosine. The numbers at the top indicate the residue number. The regions with the most polymorphic residues are marked with black squares.

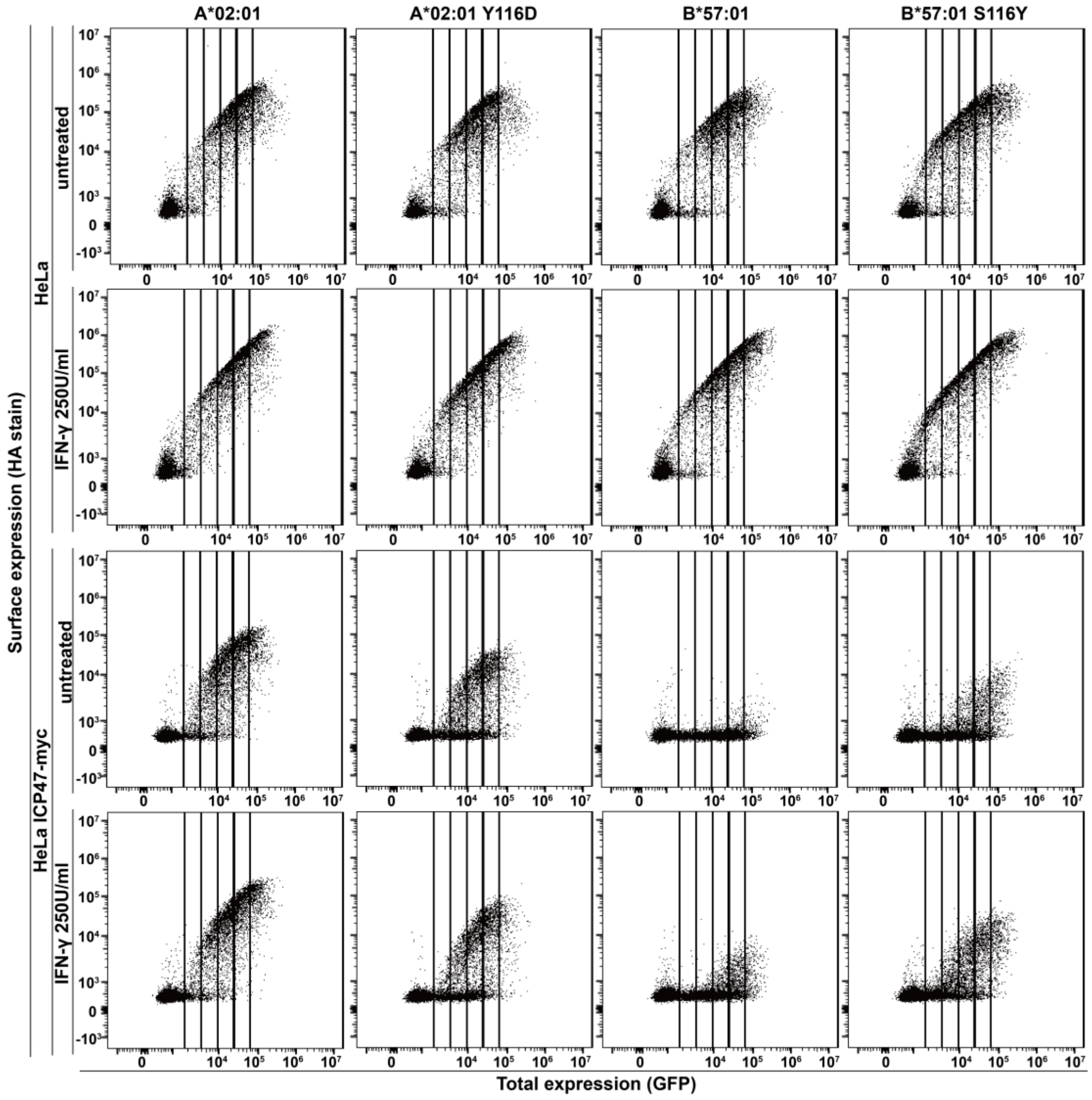

Supplementary Figure 9 corresponding to Figure 7C. The surface expression of T1D-associated A*02:01, B*57:01 WT and the corresponding mutants – all representative flow cytometry data

HeLa and HeLa ICP47-myc cells expressing HA-A*02:01-GFP, HA-B*57:01-GFP WT and the indicated mutants were stained with anti-HA antibody (clone:12CA5) to indicate HLA I allotype expression at the cell surface. The total expression is shown by GFP signal. The area of the scatter plots indicating GFP (x-axis) versus HA (y-axis) fluorescence was divided into several vertical sectors. D, aspartic acid; S, serine; Y, tyrosine.

References

1. Stranzl T, Larsen MV, Lundegaard C, Nielsen M. Netctlpan: Pan-Specific Mhc Class I Pathway Epitope Predictions. *Immunogenetics* (2010) 62(6):357-68. Epub 2010/04/10. doi: 10.1007/s00251-010-0441-4.

2. James EA, Mallone R, Kent SC, DiLorenzo TP. T-Cell Epitopes and Neo-Epitopes in Type 1 Diabetes: A Comprehensive Update and Reappraisal. *Diabetes* (2020) 69(7):1311-35. Epub 2020/06/21. doi: 10.2337/dbi19-0022.
