# Supplementary material for: Differences in F pocket impact on HLA I genetic associations with autoimmune diabetes
Source: Front Immunol. 2024 Mar 25;15:1342335. doi: 10.3389/fimmu.2024.1342335 (PMC11003304; doi:10.3389/fimmu.2024.1342335)
Supplement: Supplementary file 2 [file Table_1.docx]

**Supplementary Table 1. Sequences of primers used in the study.**

| Gene |  | Sequences | References |
| --- | --- | --- | --- |
| β-actin | FP | AGCCTCGCCTTTGCCGAT | designed in this study |
|  | RP | GAATCCTTCTGACCCATGCC |  |
| TAP1 | FP | CCAAGTGGCCAGACAGTCAT | designed in this study |
|  | RP | AGAAGGCGTTCAGAAGTCCG |  |
| TAP2 | FP | AATCCCTCACTATTCTGGTCGT | primer bank ID: 73747916c2 |
|  | RP | TCGAGACATGGTGTAGGTGAAG |  |
| Tapasin | FP | CCTGGAGGTAGCAGGTCTTTC | primer bank ID: 262527240c2 |
|  | RP | ATCCTTGCAGGTGGACAGGTA |  |
| TAPBPR | FP | TGCCCGGCCTCACTATACA | PMID:37557169 |
|  | RP | GCTCAGTCGTACTTTAGGGGAAG |  |
| ERAP1 | FP | GCAAACCTTACCACGCTGAC | PMID: 24028501 |
|  | RP | GGTTCTTCCGATAGCCTCTCTC |  |
| ERAP2 | FP | GATCTTGAAATCACGAATGCCAC | primer bank ID: 194306627c2 |
|  | RP | GAGGCGTAAGTTTCTCTGGAAC |  |
| HLA-ABC | FP | CAGTTCGTGAGGTTCGACAG | PMID: 32308549 |
|  | RP | CAGCCGTACATGCTCTGGA |  |
