# Supplementary material for: Differences in F pocket impact on HLA I genetic associations with autoimmune diabetes
Source: Front Immunol. 2024 Mar 25;15:1342335. doi: 10.3389/fimmu.2024.1342335 (PMC11003304; doi:10.3389/fimmu.2024.1342335)
Supplement: Supplementary file 4 [file Table_3.docx]

**Supplementary Table 3 corresponding to Supplementary Table 2. Numbers of predicted binders to each HLA class I allotype.**

|  | **A0201** | **A2402** | **B3901** | **B3906** | **B4405** | **A1101** | **B3801** | **B4402** | **B4403** | **B5701** |
| --- | --- | --- | --- | --- | --- | --- | --- | --- | --- | --- |
| **PPI** | 12 (9) | 4 (2) | 13 (0) | 8 (1) | 1 (0) | 1 (0) | 11 (0) | 1 (0) | 1 (0) | 4 (0) |
| **GAD65** | 47 (6) | 52 (0) | 59 (0) | 44 (0) | 33 (0) | 46 (0) | 50 (0) | 40 (0) | 41 (0) | 42 (0) |
| **ZnT8** | 50 (25) | 31 (0) | 35 (0) | 39 (0) | 15 (0) | 28 (0) | 27 (0) | 19 (0) | 20 (0) | 31 (0) |
| **IGRP** | 32 (6) | 60 (0) | 34 (0) | 42 (0) | 8 (0) | 13 (0) | 39 (0) | 12 (0) | 9 (0) | 54 (0) |
| **IAPP** | 8 (2) | 3 (0) | 7 (0) | 4 (0) | 2 (0) | 9 (0) | 4 (0) | 1 (0) | 1 (0) | 3 (0) |
| **IA-2** | 70 (7) | 44 (0) | 95 (0) | 98 (0) | 64 (0) | 49 (0) | 98 (0) | 65 (0) | 67 (0) | 45 (0) |
| **CHGA** | 19 (3) | 4 (0) | 40 (0) | 25 (0) | 62 (0) | 28 (0) | 30 (0) | 45 (0) | 41 (0) | 9 (0) |
| **S100β** | 4 (1) | 2 (0) | 10 (0) | 4 (0) | 16 (0) | 1 (0) | 8 (0) | 15 (0) | 14 (0) | 5 (0) |
| **ISL1** | 23 (1) | 12 (0) | 22 (0) | 30 (0) | 13 (0) | 20 (0) | 18 (0) | 6 (0) | 5 (0) | 17 (0) |
| **UCN3** | 16 (1) | 7 (0) | 14 (0) | 15 (0) | 6 (0) | 14 (0) | 12 (0) | 7 (0) | 7 (0) | 15 (0) |
| **VDBP** | 39 (2) | 35 (0) | 43 (0) | 32 (0) | 36 (0) | 39 (0) | 33 (0) | 32 (0) | 29 (0) | 21 (0) |
| **GLIPR1** | 21 (1) | 30 (0) | 24 (0) | 29 (0) | 10 (0) | 12 (0) | 24 (0) | 11 (0) | 9 (0) | 29 (0) |
| **GFAP** | 27 (3) | 21 (0) | 57 (0) | 53 (0) | 56 (0) | 25 (0) | 47 (0) | 53 (0) | 56 (0) | 12 (0) |
| **KCNK16** | 32 (1) | 42 (0) | 20 (0) | 24 (0) | 21 (0) | 11 (0) | 24 (0) | 20 (0) | 21 (0) | 33 (0) |
| **KIF1A** | 92 (1) | 132 (0) | 163 (0) | 178 (0) | 149 (0) | 130 (0) | 147 (0) | 148 (0) | 141 (0) | 86 (0) |
| **PCSK2** | 33 (1) | 39 (0) | 51 (0) | 51 (0) | 44 (0) | 38 (0) | 49 (0) | 47 (0) | 46 (0) | 39 (0) |
| **SCG5** | 7 (1) | 7 (0) | 12 (0) | 16 (0) | 20 (0) | 8 (0) | 15 (0) | 21 (0) | 22 (0) | 14 (0) |
| **Total** | 532 (71) | 525 (2) | 699 (0) | 692 (1) | 556 (0) | 472 (0) | 636 (0) | 543 (0) | 530 (0) | 459 (0) |

The number of predicted binders from each auto-antigen to each allele is listed. The published diabetogenic antigens within the predicted binders are listed in the brackets. Abbreviations: CHGA (chromogranin A), GAD65 (glutamic acid decarboxylase 65), GFAP (glial fibrillary acidic protein), GLIPR1 (GLI pathogenesis-related 1), IA-2 (insulinoma-associated protein 2), IAPP (islet amyloid polypeptide), IGRP (islet-specific glucose-6-phosphatase catalytic subunit-related protein), ISL1 (islet-1), KCNK16 (potassium channel subfamily K member 16), KIF1A (kinesin-like protein KIF1A), PCSK2 (prohormone convertase 1), PPI (pre-pro-insulin), S100β (S100 calcium-binding protein β), SCG5 (secretogranin V), UCN3 (urocortin III), VDBP (vitamin D-binding protein), ZnT8 (zinc transporter 8).
