# Supplementary material for: Differences in F pocket impact on HLA I genetic associations with autoimmune diabetes
Source: Front Immunol. 2024 Mar 25;15:1342335. doi: 10.3389/fimmu.2024.1342335 (PMC11003304; doi:10.3389/fimmu.2024.1342335)
Supplement: Supplementary file 5 [file Table_4.docx]

**Supplementary Table 4 corresponding to Supplementary Table 2. The number of unique binders to HLA I allotypes.**

|  | **Predicted to bind to at least one HLA I molecule** | **Predicted to bind to more than one HLA I molecule** |
| --- | --- | --- |
| **PPI** | 37 | 9 |
| **GAD65** | 260 | 102 |
| **ZnT8** | 202 | 55 |
| **IGRP** | 197 | 62 |
| **IAPP** | 30 | 9 |
| **IA-2** | 391 | 158 |
| **CHGA** | 160 | 71 |
| **S100β** | 35 | 22 |
| **ISL1** | 117 | 29 |
| **UCN3** | 75 | 23 |
| **VDBP** | 206 | 74 |
| **GLIPR1** | 120 | 44 |
| **GFAP** | 217 | 106 |
| **KCNK16** | 155 | 53 |
| **KIF1A** | 767 | 322 |
| **PCSK2** | 249 | 97 |
| **SCG5** | 69 | 32 |
| **Total** | 3287 | 1268 |

Abbreviations: CHGA (chromogranin A), GAD65 (glutamic acid decarboxylase 65), GFAP (glial fibrillary acidic protein), GLIPR1 (GLI pathogenesis-related 1), IA-2 (insulinoma-associated protein 2), IAPP (islet amyloid polypeptide), IGRP (islet-specific glucose-6-phosphatase catalytic subunit-related protein), ISL1 (islet-1), KCNK16 (potassium channel subfamily K member 16), KIF1A (kinesin-like protein KIF1A), PCSK2 (prohormone convertase 1), PPI (pre-pro-insulin), S100β (S100 calcium-binding protein β), SCG5 (secretogranin V), UCN3 (urocortin III), VDBP (vitamin D-binding protein), ZnT8 (zinc transporter 8).
